# Development of a Method for Producing Nanoporous Carbon Material from Lithium Citrate

**DOI:** 10.3390/ma19214686

**Published:** 2026-11-03

**Authors:** Georgiy Aleksan, Alexander Zakharov, Zhanibek Ayaganov, Makhmut Biisenbayev, Vladimir Pavlenko

**Affiliations:** 1 The Institute of Combustion Problems, 172 Bogenbay Batyr St., Almaty 050012, Kazakhstan; 2 Carbonix Group LLP, 43 Nazarbayev Ave., Almaty 050012, Kazakhstan; 3 UNESCO Chair for Sustainable Development, Farabi University, 71 Al-Farabi Ave., Almaty 050040, Kazakhstan

**Keywords:** lithium citrate, porous carbon, lithium carbonate, surface functional groups, aqueous supercapacitor, capacitive deionization, lithium bis(trifluoromethanesulfonyl)imide

## Abstract

**Highlights:**

- Pyrolysis of lithium citrate yields Li_2_CO_3_ and Li_2_O; leaching leaves a carbon of thin, crumpled macroporous walls.
- The leached carbon has a BET area of 342 m^2^ g^−1^, with micropores giving much of the area but little of the pore volume.
- Lithium citrate alone yields carbon with 91.5 F g^−1^ in 6 mol L^−1^ KOH and 57.6 F g^−1^ in aqueous LiTFSI.
- Concentrated aqueous LiTFSI extends the working voltage to 2.0 V, with reproducible behavior in three coin cells.
- Batch CDI over 100 cycles shows stable charge recovery; charge efficiency is the property to improve for desalination.

**Abstract:**

Porous carbon was prepared by direct carbonization of tri-lithium citrate tetrahydrate at 800 °C, followed by acid leaching and re-annealing. Pyrolysis produced Li_2_CO_3_ and Li_2_O within the carbonizate; their removal left an oxygen-functionalized disordered carbon with an openwork structure of crumpled sheets, a BET area of 342 m^2^ g^−1^ and micropores near 0.9 nm that supply about 40% of the NLDFT surface area. In a symmetric cell with 6 mol L^−1^ KOH, the carbon delivered a single-electrode capacitance of 91.5 F g^−1^ at 0.5 A g^−1^ and retained 80.2 F g^−1^ at 5 A g^−1^, with nearly rectangular voltammograms and coulombic efficiencies of at least 98.9%. In concentrated aqueous lithium bis(trifluoromethanesulfonyl)imide (10 mol kg^−1^), three symmetric coin cells extended the voltage window to 2.0 V and gave a reproducible capacitance of 57.6 ± 0.2 F g^−1^ at 0.2 A g^−1^. In batch capacitive deionization with 10 mmol L^−1^ NaCl at 1.2 V, the electrodes returned a stable charge of 1.4–1.5 C per cycle over 100 cycles and removed 2.54 ± 0.28 mg g^−1^ of salt in the first five cycles; the decline in salt adsorption on cycling identifies charge efficiency as the next property to improve.

## 1. Introduction

Porous carbons are widely used in electrochemical energy storage and ion electrosorption, including the recovery of gold from chloride solutions [1] and the removal of heavy metals by membrane capacitive deionization [2], because their surface area, pore connectivity, and surface chemistry can be adjusted through synthesis. Chemical activation with KOH is an established route to high surface areas: reactions between potassium-containing species and the carbon framework generate micropores, while increasing temperature or activator loading can widen the pores [3,4]. These benefits are accompanied by carbon consumption, handling of a corrosive reagent, and subsequent washing. Hard templating provides a complementary route in which an inorganic phase occupies space during carbon formation and is then dissolved [5,6]. The central preparation problem is therefore to obtain accessible storage pores and transport pathways without adding unnecessary mixing and template-preparation steps.

Organic metal salts combine a carbon-containing anion and a metal cation in one precursor. They can serve as self-activating carbon sources or as activators of an additional precursor [4,7,8,9]. Carbonate and oxide formation, gas evolution, and reactions with the carbon framework govern pore development. Shi et al. review these routes in terms of activation, self-activation, and control of carbon architecture [8], while their CDI-focused review relates the pore structure to ion-electrosorption performance [10]. Single-source precursors simplify the distribution of inorganic species within the carbonizing matrix and the subsequent generation of porosity.

Citrate salts illustrate the influence of precursor chemistry. Direct carbonization of potassium citrate produces interconnected porous carbons, whereas calcium-, zinc-, and iron-containing citrates form mesoporous carbons through inorganic intermediates [6,7,11]. Recent CDI studies use citrate in multicomponent formulations. Shi et al. combined potassium citrate and sodium nitrate to activate phoenix-tree leaves, obtaining an N-doped carbon with a BET area of 1912.2 m^2^ g^−1^ and measured NaCl electrosorption [12]. Miao et al. combined glucose, NaCl, and potassium citrate at 800 °C to prepare electrodes for ammonium removal [13]. These formulations use citrate alongside a separate carbon source or salt template, allowing activation and templating to be adjusted independently.

Li et al. demonstrated direct carbonization of tri-lithium citrate tetrahydrate at 650 °C for 2 h under argon, followed by dilute HCl, water, and ethanol washing, producing porous LiC with a BET area of 222.5 m^2^ g^−1^ [14]. Their alkali–citrate carbons were evaluated as potassium-ion battery anodes. Lithium citrate has also been combined with sucrose to prepare porous carbon hosts [15,16]. In situ high-temperature XRD by Deng et al. showed the formation of Li_2_CO_3_ followed by Li_2_O during alkali–citrate conversion [17], and studies of alkali–metal coordination precursors further demonstrated cation-dependent carbon textures [18]. These findings motivate examination of lithium-citrate conversion at higher temperatures and the resulting carbon in aqueous capacitive cells.

Li_2_CO_3_ melts near 720 °C [19], below the 800 °C carbonization temperature used here. Thermal and carbothermic conversion can also generate Li_2_O [20], producing carbonate melt and solid inorganic intermediates within the carbonizing precursor. Salt-melt synthesis provides a basis for understanding their role in pore formation [21,22]. Conversion of all lithium in anhydrous tri-lithium citrate into Li_2_CO_3_ corresponds to 1.5 mol of carbonate per mole of citrate, approximately 53% of the precursor mass. The large inorganic fraction can therefore contribute substantially to the carbon architecture. Since porous lithium-citrate carbon also forms at 650 °C [14], pore development involves precursor decomposition and gas evolution as well as the possible contribution of molten carbonate at 800 °C.

The balance between microporous storage surface and wider transport pores is central to aqueous energy storage and capacitive deionization (CDI) [10,23,24,25,26,27,28,29]. Micropores supply adsorption sites, while mesopores and macropores provide pathways through the electrode. Citrate-assisted CDI studies illustrate how this architecture supports ion electrosorption under defined electrolyte and cell conditions [12,13]. Characterizing the pore network together with the charging response is therefore necessary to assess the function of a new citrate-derived carbon.

Here, tri-lithium citrate tetrahydrate is carbonized at 800 °C, acid-leached, and re-annealed at 800 °C without an added carbon source, template, or activator. The selected temperature permits carbonate melting during carbonization. Thermal analysis and diffraction of the carbonizate are combined with XRD, Raman, FTIR, and nitrogen adsorption of the final carbon. Three independently assembled symmetric coin cells in aqueous 10 mol kg^−1^ lithium bis(trifluoromethanesulfonyl)imide (LiTFSI) establish its capacitance, rate response, and impedance over 0–2.0 V. One hundred batch CDI cycles in 10 mmol L^−1^ NaCl then quantify its salt adsorption, charge efficiency, and cycling behavior. The study relates this preparation route to the resulting pore structure, surface functionalities, aqueous charging, and desalination behavior.

## 2. Materials and Methods

### 2.1. Materials

Tri-lithium citrate tetrahydrate (Li_3_C_6_H_5_O_7_·4H_2_O; Merck KGaA, Darmstadt, Germany) was used as received. Lithium bis(trifluoromethanesulfonyl)imide (LiTFSI) and a polytetrafluoroethylene (PTFE) dispersion were purchased from Sigma-Aldrich (Merck KGaA, Darmstadt, Germany), and sodium chloride from Laborpharma (Almaty, Kazakhstan). Hydrochloric acid of analytical grade (Labkhimprom, Almaty, Kazakhstan) was used for leaching. Aqueous solutions were prepared with deionized water (18.2 MΩ cm).

### 2.2. Carbonization, Leaching, and Re-Annealing

The salt (20 g) was heated in a horizontal quartz tube reactor under a nitrogen flow of 200 cm^3^ min^−1^ at 10 °C min^−1^ to 250 °C, held for 30 min, and heated at the same rate to 800 °C and held for 1 h. The carbonizate is denoted Li-C. It was leached in 2 mol L^−1^ HCl at 60 °C for at least 12 h under stirring, vacuum-filtered, washed with deionized water to neutral pH, dried under vacuum at 110 °C for 24 h, ground and sieved, and re-annealed at 800 °C for 1 h under nitrogen. The final carbon is denoted Li-CL.

### 2.3. Characterization

Thermogravimetry with differential thermal analysis (TG-DTA) of the salt was performed on an STA 449 F3 instrument (NETZSCH-Gerätebau GmbH, Selb, Germany) in an Al_2_O_3_ crucible, from 30 to 950 °C at 10 °C min^−1^ under argon. The DTG curve was obtained from the mass curve smoothed with a Savitzky–Golay filter (window 21 °C, second order).

Powder X-ray diffraction (XRD) was recorded on a TD-3700 diffractometer (Dandong Tongda Science & Technology, Dandong, China) with a MYTHEN2R detector (DECTRIS, Baden-Dättwil, Switzerland) in Bragg–Brentano geometry using Cu Kα radiation and a Ni filter. The tube voltage was 45 kV for Li-C and 40 kV for Li-CL, at 40 mA. Scans covered 5–60° 2θ with a step of 0.0188° and 0.6 s per point. The Li-C profile was analyzed in Jana2006 (version 20/02/2023; Institute of Physics of the Czech Academy of Sciences, Prague, Czech Republic) by Le Bail decomposition over 10–60° 2θ, with a five-term Legendre background and a pseudo-Voigt profile including axial-divergence asymmetry. The profile and refinement parameters are given in Figure S1 and Table S1.

Raman spectra of Li-CL were recorded at four locations on a Solver Spectrum instrument (NT-MDT Spectrum Instruments, Zelenograd, Russia) with a 473 nm laser at 4 cm^−1^ resolution. The 900–1800 cm^−1^ region was fitted with a linear background and a four-band model comprising Lorentzian D4, D1, and G profiles and a Gaussian D3 profile [30]. The choice of this model is discussed in Section 3.2 and the Supplementary Information.

Fourier-transform infrared (FTIR) spectra were recorded on a Bruker ALPHA II spectrometer (Bruker Optik, Ettlingen, Germany) equipped with a platinum single-reflection diamond attenuated total reflection (ATR) module over 4000–400 cm^−1^ at a resolution of 4 cm^−1^, with 24 co-added scans. Li-CL powder was mixed with KBr (Shanghai Macklin Biochemical Co., Ltd., Shanghai, China) at a mass ratio of 1:100 and measured on the ATR crystal; no pellet was pressed.

The morphology of Li-CL was examined by scanning electron microscopy (SEM) on an SEM5000Pro microscope (CIQTEK, Hefei, China) with the in-lens secondary electron detector at an accelerating voltage of 15 kV, a working distance of 9.8 mm, and magnifications of 1000–30,000×. Scale bars were drawn from the horizontal field width recorded by the instrument. Energy-dispersive X-ray (EDX) spectra and elemental maps of Li-CL were acquired on the same microscope at 20 kV with an X-Plore 30 silicon drift detector (Oxford Instruments, High Wycombe, UK) on powder mounted on carbon adhesive tape; acquisition details are given in the Supplementary Information.

Nitrogen isotherms were measured at 77 K on a 3Flex analyzer (Micromeritics, Norcross, GA, USA) after degassing at 350 °C for 12 h. The BET area was calculated in the pressure range selected with the Rouquerol consistency criteria [31,32]. The adsorption-equivalent liquid volume was obtained by the Gurvich conversion at the highest measured adsorption pressure, p/p_0_ = 0.9833 [33]. Pore size distributions were taken from the adsorption-branch heterogeneous-surface two-dimensional NLDFT model for carbon slit pores (HS-2D-NLDFT) [34], with non-negative regularization of 0.01. Micropore quantities at 2 nm were interpolated from the cumulative distribution. The report of the 3Flex software (version 6.03) also provides adsorption-branch BJH analysis with a Harkins–Jura thickness curve and Faas correction; it is used only for comparison with NLDFT in the mesopore range [33].

### 2.4. Electrochemical Measurements

Free-standing electrodes contained 85 wt% Li-CL, 10 wt% carbon black (Super C45, Imerys Graphite & Carbon, Bodio, Switzerland), and 5 wt% PTFE. Three symmetric coin cells were assembled using 12 mm electrode discs, SS316 current collectors, and ashless cellulose filter-paper separators (red-ribbon grade; Laborpharma, Almaty, Kazakhstan). Each cell contained 200 µL of aqueous 10 mol kg^−1^ LiTFSI. The electrodes were 100 µm thick, with a composite mass loading of about 4.7 mg cm^−2^ (4.0 mg cm^−2^ of Li-CL). Current density was normalized by half of the total Li-CL mass of the cell, following the symmetric-cell convention.

Cyclic voltammetry (CV) and galvanostatic charge–discharge (GCD) were recorded with a CorrTest potentiostat (Wuhan Corrtest Instruments, Wuhan, China) and CSStudio software (version 6.6) over 0–2.0 V at 2–200 mV s^−1^ and 0.2–10 A g^−1^, respectively. Values are reported as the mean ± standard deviation of the three cells. The single-electrode specific capacitance was calculated from the delivered discharge charge *Q*_d_ [35]:

(1) $$C_{s}\;=\;\frac{4\;Q_{d}}{m\;\Delta U}\;$$

where *m* is the total Li-CL mass in both electrodes and Δ*U* = 2.0 V is the nominal cell window. This definition includes the loss of usable voltage caused by the current-reversal step. Capacitances calculated from the post-step discharge span are reported separately in Table S2. Coulombic efficiency was obtained from the discharge-to-charge integral ratio. Discharge energy was calculated by integrating *U*(*t*)|*I*(*t*)| over the positive-voltage discharge and normalizing by the active mass in both electrodes.

Electrochemical impedance spectroscopy (EIS) was measured after CV/GCD with a VSP potentiostat (Bio-Logic, Seyssinet-Pariset, France) at the acquired open-circuit potential following a 120 s hold. A nominal 10 mV perturbation was applied from 500 kHz to 10 mHz with ten frequencies per decade. Complex capacitance was calculated from 1/(j*ωZ*), with the symmetric-cell factor 4/*m*. The relaxation time was taken as 1/*f* at the loss-capacitance maximum [36].

The voltage window was verified in a separate CV series, recorded after the rate measurements, at 2 mV s^−1^ with the upper cutoff increased from 1.8 to 2.4 V in 0.1 V steps (Figure S2).

A symmetric two-electrode cell with 6 mol L^−1^ KOH was assembled from electrodes of the same composition (1.13 cm^2^, 4.21 mg of Li-CL per electrode) with stainless steel 316 current collectors and a hydrophilic PTFE separator (Deschem, Changshu, China) and tested with a VSP potentiostat over 0–1.0 V by CV at 5–50 mV s^−1^, GCD at 0.5–5 A g^−1^ (referred to the active mass of one electrode), and EIS at 0 V from 300 kHz to 10 mHz with a 5 mV amplitude. For this cell, the capacitance was calculated from the discharge time after the ohmic drop, *C*_s_ = 4*I*Δ*t*/(*m*Δ*U*), where Δ*U* is the voltage change after the drop; values are medians of the last three of eleven cycles [35].

### 2.5. Capacitive Deionization Measurements

Two free-standing electrodes with the composition given in Section 2.4 (85 wt% Li-CL, 10 wt% carbon black, 5 wt% PTFE), 5 cm in diameter and 100 µm thick (combined Li-CL mass *m* = 163.9 mg), were assembled in a flow-by CDI cell with titanium current collectors and a nylon spacer. The cell was operated in batch mode with 100 mL of 10.0 mmol L^−1^ NaCl recirculated at 14 mL min^−1^ between the cell and a closed reservoir. After an initial equilibration of 10–15 min without applied voltage, 100 cycles were recorded, each comprising 900 s at 1.2 V (adsorption) and 900 s at 0 V (regeneration). The voltage steps were applied with a CorrTest CS310X potentiostat and CSStudio software. The conductivity of the solution in the reservoir was logged every 10 s with a Bante 900 multiparameter meter (Bante Instruments, Shanghai, China) and a CON-1 electrode with temperature compensation. Time zero was set at the first 1.2 V step.

The conductivity modulation was superimposed on a gradual upward drift. The drift was removed by subtracting a shape-preserving piecewise cubic Hermite baseline through the mean conductivity of each cycle, assigned to the cycle midpoint; for presentation, the corrected conductivity is referenced to the initial baseline value of 936 µS cm^−1^. For each cycle, the conductivity change Δ*κ* was taken as the difference between the mean baseline-corrected conductivity at 1100–1650 s (regeneration plateau) and at 250–800 s (adsorption plateau). Δ*κ* was converted into the concentration change Δ*C* assuming conductivity proportional to NaCl concentration. The salt adsorption capacity (SAC) and charge efficiency Λ were calculated as follows [23,25]:

(2) $$\mathrm{SAC}\;=\;\frac{\Delta C\;V\;M_{\mathrm{NaCl}}}{m}\;$$

(3) $$\Lambda =\frac{F\;\cdot \;\mathrm{SAC}\;\cdot \;m}{M_{\mathrm{NaCl}}\;Q}\;$$

where *V* = 0.100 L, *M*_NaCl_ = 58.44 g mol^−1^, *F* is the Faraday constant, SAC is expressed in g g^−1^ in Equation (3), and *Q* is either the charge passed at 1.2 V (*Q*_ch_) or the charge returned at 0 V (*Q*_dis_). Both charges were obtained by integrating the recorded current over the 900 s steps. The coulombic efficiency was calculated as *Q*_dis_/*Q*_ch_. A nominal single-electrode capacitance was obtained from Equation (1) with *Q*_d_ = *Q*_dis_ and Δ*U* = 1.2 V.

## 3. Results and Discussion

### 3.1. Thermal Conversion of Lithium Citrate

The precursor loses 25.2% of its initial mass by 250 °C, close to the 25.6% water content calculated for Li_3_C_6_H_5_O_7_·4H_2_O (Figure 1). The main dehydration event has a DTG minimum near 116 °C and a DTA extremum near 125 °C. Decomposition of the anhydrous precursor proceeds through overlapping mass-loss events between about 280 and 600 °C. The remaining mass is 40.9% at 600 °C and 37.3% at 700 °C; conversion of the tetrahydrate to Li_2_CO_3_ alone would leave 39.3%. In Figure 1a, dashed lines mark the residues calculated for the anhydrous salt (74.4%), Li_2_CO_3_, and Li_2_O; in the DTA curve (Figure 1c), exothermic effects point downward.

An exothermic event near 608 °C accompanies substantial mass loss. The residue at 700 °C is already below the carbonate-only value, so the mass loss is not limited to conversion into Li_2_CO_3_. In the tube-reactor synthesis, the carbonizate yield of 34.3% of the precursor mass is likewise below the 39.3% expected for Li_2_CO_3_ alone, in agreement with the partial conversion to Li_2_O observed by XRD. Acid leaching left 1.99% of the precursor mass as carbon (5.8% of the mass of Li-C before re-annealing).

The sharp endothermic peak at 725 °C agrees with the melting point of Li_2_CO_3_ [19]. Earlier thermal studies likewise report carbonate formation during lithium-citrate decomposition [17,37]. Above 700 °C, further mass loss reduces the residue to 13.1% at 950 °C. Both thermal decomposition of Li_2_CO_3_ and the carbothermic reaction Li_2_CO_3_(l) + C(s) → Li_2_O(s) + 2CO(g) can produce Li_2_O [20]. Together with the Li_2_CO_3_/Li_2_O diffraction pattern, these events support carbonate formation followed by melting and partial oxide formation during the 800 °C treatment. The 950 °C residue is lower than the 15.9% calculated for Li_2_O alone, so additional mass loss occurs at the highest temperatures.

### 3.2. Phase Composition and Vibrational Characterization

The carbonizate contains monoclinic Li_2_CO_3_ and cubic Li_2_O (Figure 2a). The Le Bail analysis gives a = 8.3665(5) Å, b = 4.9793(3) Å, c = 6.2014(3) Å, and β = 114.6899° (fixed) for Li_2_CO_3_ (C2/c), and a = 4.61716(19) Å for Li_2_O (Fm-3m). Reflections near 33.6, 39.0, and 56.3° 2θ support the Li_2_O assignment. The full profile, including the tested LiOH candidate, gives R_p_ = 4.19%, R_wp_ = 6.14%, and GOF = 2.76 (Figure S1 and Table S1).

After acid leaching and re-annealing, the sharp Li_2_CO_3_ and Li_2_O reflections disappear, while broad features near 24 and 43° 2θ remain (Figure S3). Removal of the crystalline inorganic phases leaves a predominantly disordered carbon product.

The ATR-FTIR spectrum contains a carbonyl band near 1730 cm^−1^ and C–O stretching bands near 1273 and 1072 cm^−1^ (Figure S4) [38]. The broad band near 1566 cm^−1^ includes aromatic C=C skeletal vibrations and a possible contribution from conjugated carbonyl groups [38,39]. The feature near 2921 cm^−1^ is assigned to aliphatic C–H stretching. These bands identify oxygen-containing functionalities in the final Li-CL; their assignments are summarized in Table S3.

The Raman spectra show broad D and G bands characteristic of disordered carbon [40]. Two-Voigt fits [41] give D1 and G centers of 1373.0 ± 1.3 and 1587.9 ± 0.9 cm^−1^, respectively, and a D1/G height ratio of 1.12 ± 0.05 across four locations; the four-band model gives 1.06 ± 0.21 (Table S4). The four-band model provides the lowest BIC at every location, reducing the RMSE from 5.4–10.3 to 3.4–5.6 counts (Table S4 and Figure S5). Figure 2b illustrates the four-band fit at location 3. Adding D2 gives only a small residual improvement, with its center reaching the lower bound of 1605 cm^−1^ in all four five-band fits. The four-band description therefore captures the broad carbon envelope without imposing an unstable G/D2 separation. In this model, the D1/G height ratio ranges from 0.87 to 1.34 between locations. At locations 3 and 4, the D4 width reaches its upper bound of 400 cm^−1^ (Supplementary Information), so part of this spread reflects covariance between D4 and D1. Because the mean ratio depends on the band model (1.12, 1.06, and 1.58 for two, four, and five bands), it is not converted to a crystallite size [40].

### 3.3. Morphology of the Leached Carbon

Li-CL consists of irregular particles from a few micrometers to several tens of micrometers with rough, folded surfaces (Figure 3a). At higher magnification, the particle surfaces show a porous texture with openings of up to about 2 µm (Figure 3b). At 30,000×, thin, crumpled carbon walls enclose open cells, mostly about 50–200 nm across, which form a connected macroporous network (Figure 3c) [33]. These walls are consistent with carbon formation around inorganic domains followed by their removal during leaching. Nitrogen adsorption resolves the finer pores within these walls. Local EDX spectra were dominated by C and O (Figure S6 and Table S5). Oxygen was detected in the carbon-rich regions, complementing the FTIR evidence for oxygen-containing functionalities.

### 3.4. Pore Structure

The nitrogen isotherm combines low-pressure uptake with a broad hysteresis loop and a continuous rise near saturation (Figure 4a). The adsorption branch has a type-II-like character and H3 hysteresis, consistent with the sheet-like aggregates seen by SEM [33]. The BET area is 342 m^2^ g^−1^, with C = 52.8. The adsorption-equivalent liquid volume reaches 0.928 cm^3^ g^−1^ at p/p_0_ = 0.9833. Its continued increase at high pressure indicates a substantial contribution from large voids between sheets. Key textural parameters are collected in Table 1.

HS-2D-NLDFT analysis gives a surface area of 253 m^2^ g^−1^ and a modeled pore volume of 0.890 cm^3^ g^−1^ (Figure 4b). The micropore volume below 2 nm is 0.0565 cm^3^ g^−1^, and the micropore surface area is approximately 101 m^2^ g^−1^. Micropores thus supply about 40% of the modeled surface area, although their volume is only 6.1% of the adsorption-equivalent liquid volume. The micropore maximum lies near 0.9 nm (inset in Figure 4b), while the wider-pore distribution is broad and has a volume-weighted median of about 22 nm. The terminal NLDFT bin at 50.6 nm contributes 0.0600 cm^3^ g^−1^ and includes the upper boundary of the modeled range.

The adsorption-branch BJH distribution also shows a large wide-pore contribution (Figure 4c). Both distributions are plotted per unit pore width or diameter on linear axes. The cumulative BJH volume is 0.907 cm^3^ g^−1^ over 2.05–116.87 nm, with 0.455 cm^3^ g^−1^ above 37.18 nm. BJH and NLDFT use cylindrical and slit-pore geometries, respectively, but both identify the dominance of wider pores in the pore volume [33]. The BET area exceeds the 222.5 m^2^ g^−1^ reported for lithium-citrate carbon prepared at 650 °C [14]. That study used a different washing sequence and degassing at 150 °C for 8 h (350 °C for 12 h here), so the difference cannot be attributed to the carbonization temperature alone (Table S6).

The microporous area and open wider-pore network provide complementary storage and transport regions. For dilute 1:1 electrolytes at 25 °C, the Debye length is approximately λ_D_ = 0.304/√c nm, with c in mol L^−1^ [42]. Twice this length is 19.2, 6.1, and 1.9 nm at 1, 10, and 100 mmol L^−1^; approximately 90%, 73%, and 38% of the NLDFT surface area lies below these respective widths (vertical lines in Figure 4d). At the CDI feed concentration of 10 mmol L^−1^, most of the surface therefore lies in pores narrower than twice the Debye length. The dilute-solution estimate does not apply to 10 mol kg^−1^ LiTFSI.

### 3.5. Electrochemical Behavior in Aqueous Electrolytes

In 6 mol L^−1^ KOH over 0–1.0 V, the voltammograms of Li-CL are close to rectangular without redox peaks, and the GCD curves are nearly linear and symmetric (Figure S7). The capacitance decreases from 91.5 F g^−1^ at 0.5 A g^−1^ to 80.2 F g^−1^ at 5 A g^−1^ (88% retention), and the coulombic efficiency is 98.9% at 0.5 A g^−1^ and above 99.4% at higher current densities. The ohmic drop at current reversal increases from 3.0 to 24.7 mV, corresponding to 0.72–0.59 Ω, and the loss-capacitance maximum gives a relaxation time of 1.5 s. These values were obtained with one cell. The capacitance of 91.5 F g^−1^ corresponds to 27 µF cm^−2^ of BET and 36 µF cm^−2^ of NLDFT surface; such high areal values suggest a contribution from the oxygen-containing surface groups (Section 3.2) or from ultramicropores that nitrogen probes incompletely at 77 K [33]. Because the usable voltage in KOH is limited to about 1 V by water decomposition, the concentrated LiTFSI electrolyte was used to extend the window.

Voltage-window CV at 2 mV s^−1^, recorded as a separate series after the rate measurements, showed an increasing upper-voltage current and a declining return-to-forward charge ratio as the upper cutoff was raised from 1.8 to 2.4 V (Figure S2). Charge recovery was 96.83 ± 0.18% at 2.0 V and decreased to 92.84 ± 0.28% at 2.2 V and 79.89 ± 0.75% at 2.4 V. These results confirm 2.0 V as an appropriate upper cutoff for the rate measurements.

In the rate-characterization series, the CV responses are closely reproducible across the three coin cells (Figures S8 and S9), and the CV capacitance decreases from 63.2 ± 0.2 F g^−1^ at 2 mV s^−1^ to 35.2 ± 1.1 F g^−1^ at 200 mV s^−1^. Figure 5a shows the CVs of cell 2 normalized as 4I/(νm), where m is the combined Li-CL mass in both electrodes, and Figure 5b shows the GCD curves of the same cell at 0.2–5 A g^−1^.

The GCD specific capacitance, calculated over the full 2.0 V window, is 57.6 ± 0.2 F g^−1^ at 0.2 A g^−1^, with 98.99 ± 0.05% coulombic efficiency. It decreases to 29.6 ± 1.6 F g^−1^ at 5 A g^−1^, corresponding to 51% retention (Figure 5c and Table S2). Over the same range, the current-reversal voltage step increases from 0.028 ± 0.002 to 0.696 ± 0.057 V. Calculated over the discharge span after this step, the capacitance is 58.4 ± 0.2 F g^−1^ at 0.2 A g^−1^ and 45.3 ± 0.8 F g^−1^ at 5 A g^−1^ (Figure 5c); both series are means ± sample standard deviations across the three cells. Polarization therefore consumes an increasing fraction of the available voltage and limits charge delivery at high current. The integrated discharge energy is 7.61 ± 0.02 Wh kg^−1^ at 0.2 A g^−1^ and 2.54 ± 0.25 Wh kg^−1^ at 5 A g^−1^, referred to the combined active mass of both electrodes. The energy efficiency at 0.2 A g^−1^ is 82.9 ± 0.2%.

The post-test Nyquist plots contain a high-frequency arc followed by a steep low-frequency branch (Figure 5d; the inset enlarges the high-frequency region). The high-frequency real-axis intercepts are 2.95–5.74 Ω. The real component of the single-electrode complex capacitance reaches 44.3–45.3 F g^−1^ at approximately 10 mHz, and the loss-capacitance maxima give relaxation times of 12.6–15.8 s (Figure S10). The open-circuit voltages of the cells during EIS were 0.339–0.365 V.

### 3.6. Capacitive Deionization in NaCl

Figure 6a shows the baseline-corrected solution conductivity during CDI cycling. Each 1.2 V step lowers the conductivity, and each 0 V step restores it, following the period of the applied voltage program. The conductivity change between the regeneration and adsorption plateaus averages 6.7 ± 1.1 µS cm^−1^ over cycles 5–10 and decreases to 1.3 ± 0.8 µS cm^−1^ over cycles 85–90 (insets in Figure 6a). The SAC reaches 2.95 mg g^−1^ in cycle 3 and averages 2.54 ± 0.28 mg g^−1^ over cycles 1–5 (mean ± sample standard deviation over cycles; Figure 6b and Table S7). It decreases to 1.78 ± 0.09 mg g^−1^ over cycles 16–20, 0.68 ± 0.26 mg g^−1^ over cycles 40–60, and 0.51 ± 0.30 mg g^−1^ over cycles 80–100. In the later cycles, Δ*κ* is only 1–2 µS cm^−1^, comparable to the 1 µS cm^−1^ resolution of the conductivity record, which accounts for most of the cycle-to-cycle scatter.

The conductivity transients of cycles 10–50 retain the same shape, with a decrease during the 1.2 V step and recovery at 0 V, while their amplitude diminishes (Figure 7a). The corresponding current transients nearly coincide (Figure 7b). The charge returned at 0 V averages 1.44 ± 0.05, 1.51 ± 0.02 and 1.49 ± 0.03 C over cycles 1–20, 40–60 and 80–100 (Table S7), and the nominal single-electrode capacitance is 30 ± 1 F g^−1^ over all 100 cycles. This value is lower than the 57.6 F g^−1^ obtained in 10 mol kg^−1^ LiTFSI, consistent with the much lower ionic strength of the CDI feed. The decline in SAC therefore occurs without a corresponding loss of recoverable capacitive charge.

The charge passed at 1.2 V exceeds the returned charge in every cycle. The coulombic efficiency increases from 33 ± 4% in cycles 1–5 to 62 ± 2% in cycles 80–100, while the current at the end of the charging step decreases from 3.55 to 1.33 mA (Figure 7d). The two quantities change together throughout cycling and are linearly correlated (r = −0.97 over 100 cycles), indicating that the non-recovered charge is carried by a steady faradaic current at 1.2 V. The charge efficiency based on Q_ch_ is 0.17 ± 0.02 over cycles 1–20 and falls to 0.07 ± 0.03 and 0.06 ± 0.03 over cycles 40–60 and 80–100; the corresponding values based on Q_dis_ are 0.40 ± 0.08, 0.12 ± 0.05 and 0.09 ± 0.05. The fraction of the capacitive charge that produces net salt removal thus decreases with cycling, whereas the capacitive charge itself remains nearly constant.

A decreasing charge efficiency at stable capacitance indicates a growing co-ion contribution to the electrical double layer. Such behavior is commonly associated with oxidation of the positive electrode during prolonged operation at 1.2 V [25,43]. The oxygen-containing functionalities identified by FTIR in the as-prepared carbon (Section 3.2) are compatible with this interpretation, although changes in surface chemistry during cycling were not characterized. The pore texture also limits uptake. The microporous surface of about 101 m^2^ g^−1^ is small, and about 27% of the NLDFT surface lies in pores wider than twice the Debye length at 10 mmol L^−1^ (Section 3.4), where non-overlapping diffuse layers lower the charge efficiency [25]. Li-CL in its present form therefore combines open transport pores and stable capacitive charging with low and declining salt adsorption. A larger microporous surface and suppression of faradaic charge losses are required for CDI application [10,23,24].

## 4. Conclusions

Carbonization of lithium citrate at 800 °C produces a carbonizate dominated by Li_2_CO_3_ and Li_2_O: the carbon left after leaching accounts for only 5.8% of the carbonizate mass and 1.99% of the precursor mass. Removal of the inorganic phases gives a carbon in which wide pores formed between thin walls dominate the pore volume, while micropores near 0.9 nm supply about 40% of the surface area. In alkaline electrolyte, the capacitance per unit NLDFT surface (36 µF cm^−2^) points to an additional contribution of oxygen-containing groups or of narrow micropores that nitrogen does not fully probe. In concentrated aqueous LiTFSI, the same texture supports reproducible charging up to 2.0 V; at high current, the capacitance is limited mainly by the voltage step at current reversal, since the capacitance calculated after this step retains 45.3 F g^−1^ at 5 A g^−1^. In batch CDI with 10 mmol L^−1^ NaCl at 1.2 V, the salt adsorption capacity decreases from 2.5 ± 0.3 to 0.5 ± 0.3 mg g^−1^ over 100 cycles while the returned charge remains nearly constant. The low charge efficiency and its link to the end-of-charge current identify faradaic charge loss and the limited microporous surface as the factors to address for desalination. The preparation yields a carbon combining a microporous surface with open transport pores and reproducible aqueous capacitive behavior.

## Figures and Tables

**Figure 1:**
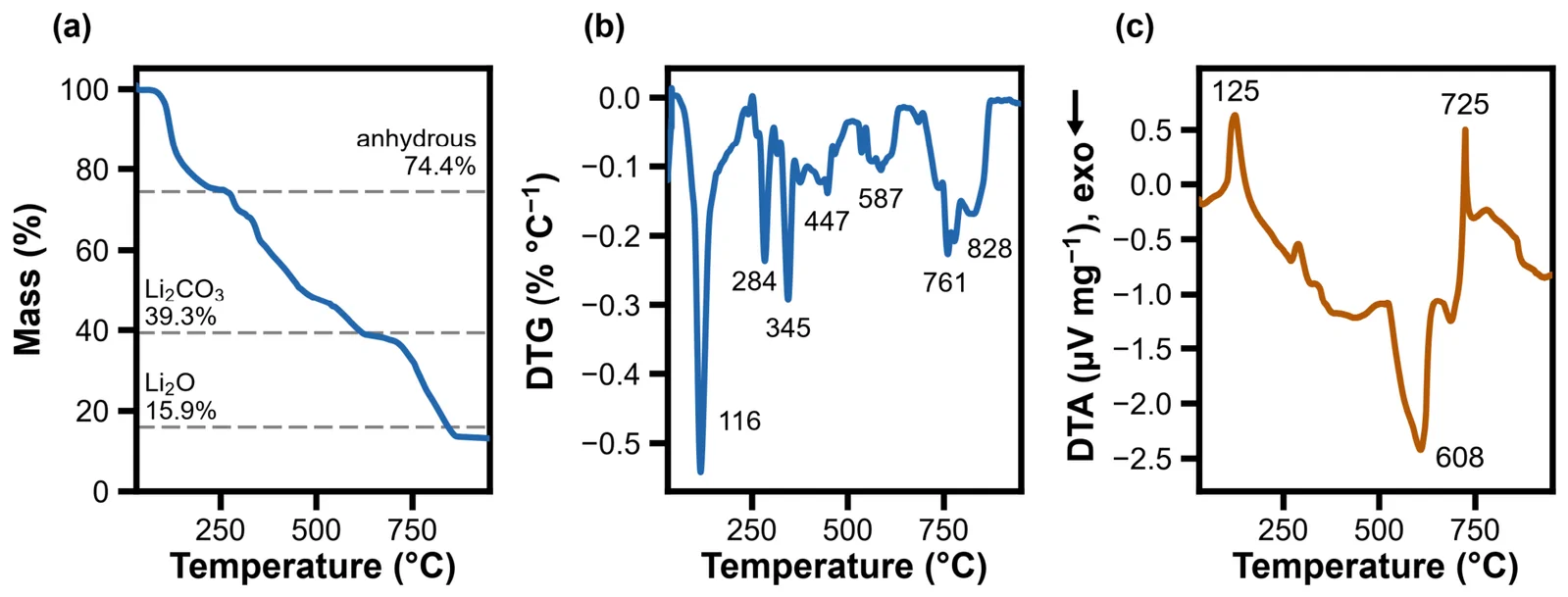
Thermal analysis of Li_3_C_6_H_5_O_7_·4H_2_O: (**a**) TG; (**b**) DTG; (**c**) DTA.

**Figure 2:**
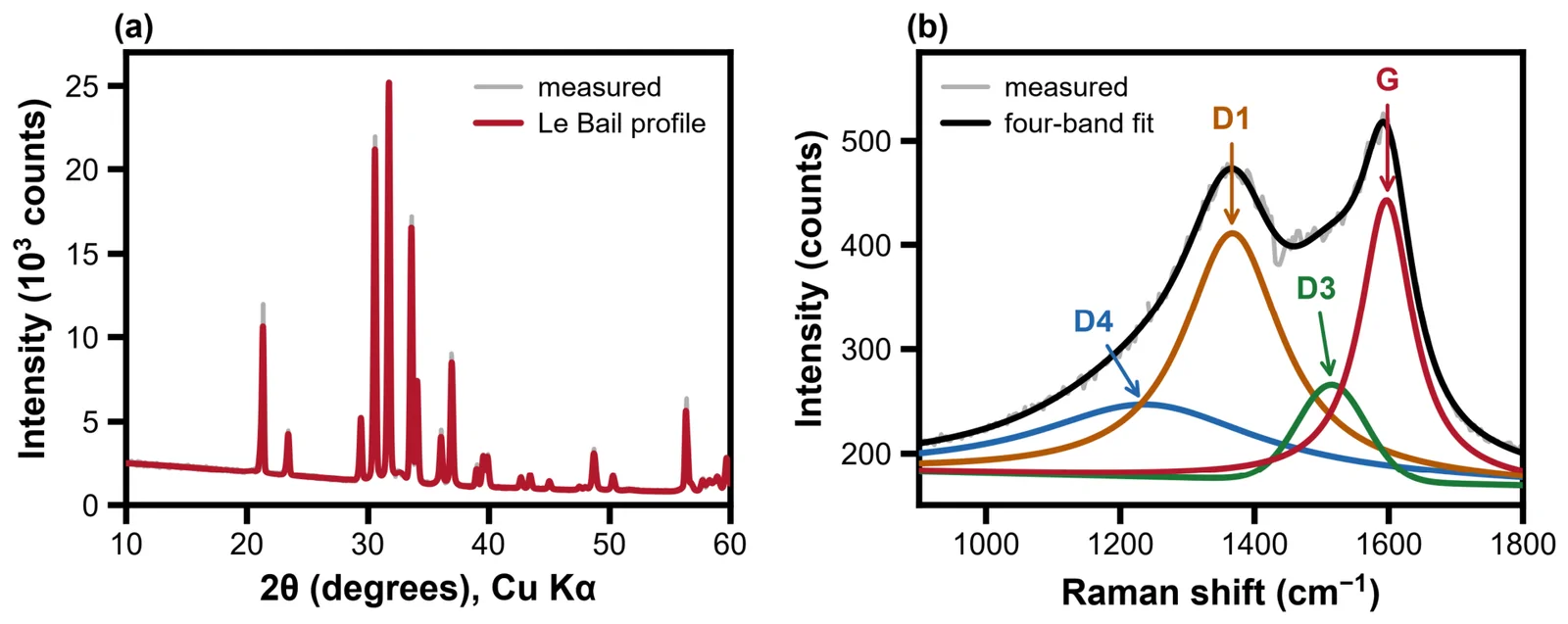
(**a**) XRD pattern of Li-C with the Le Bail profile; (**b**) Raman spectrum of Li-CL with the four-band fit.

**Figure 3:**
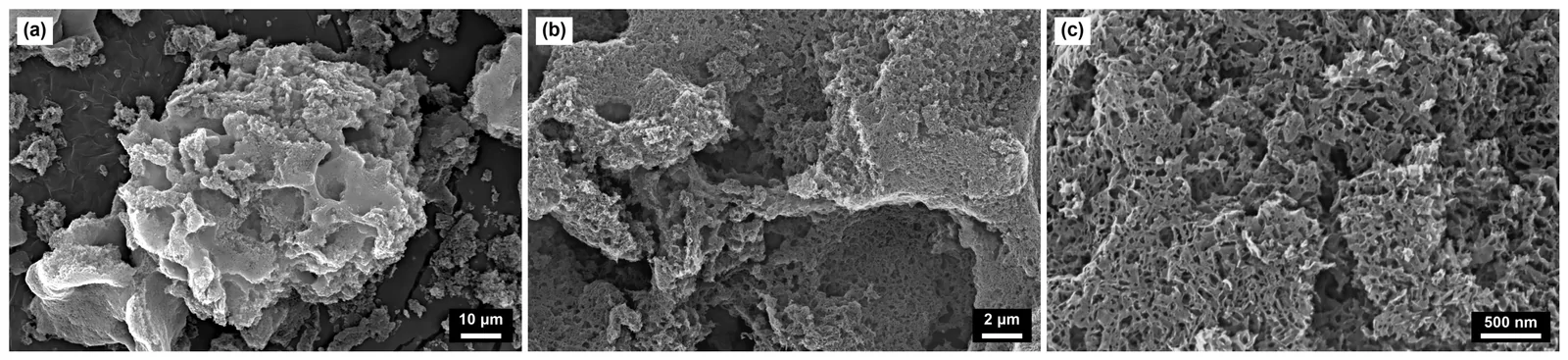
SEM images of Li-CL at (**a**) 1000×, (**b**) 5000×, and (**c**) 30,000×.

**Figure 4:**
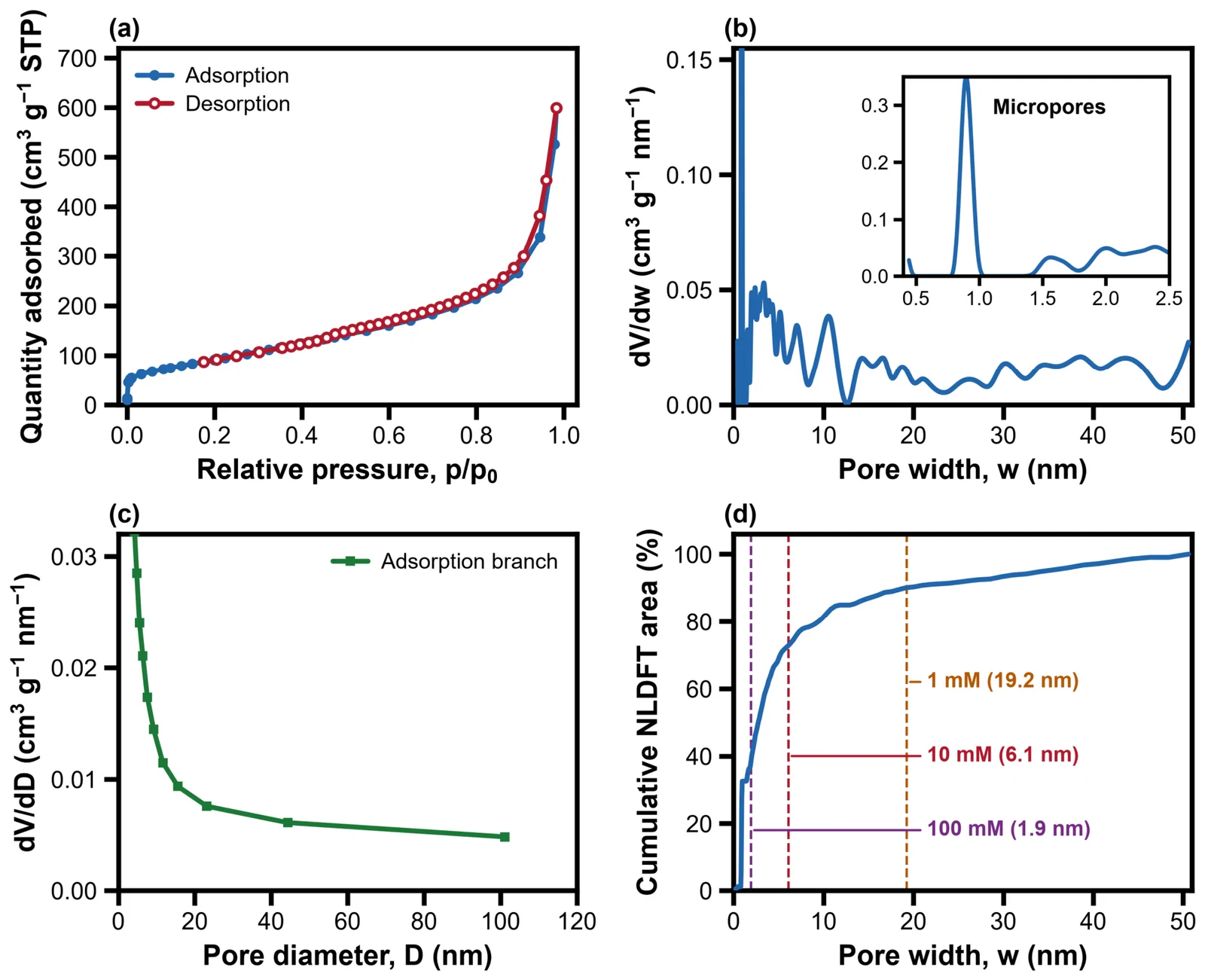
Nitrogen adsorption on Li-CL at 77 K: (**a**) isotherm; (**b**) NLDFT and (**c**) BJH pore size distributions; (**d**) cumulative NLDFT surface area.

**Figure 5:**
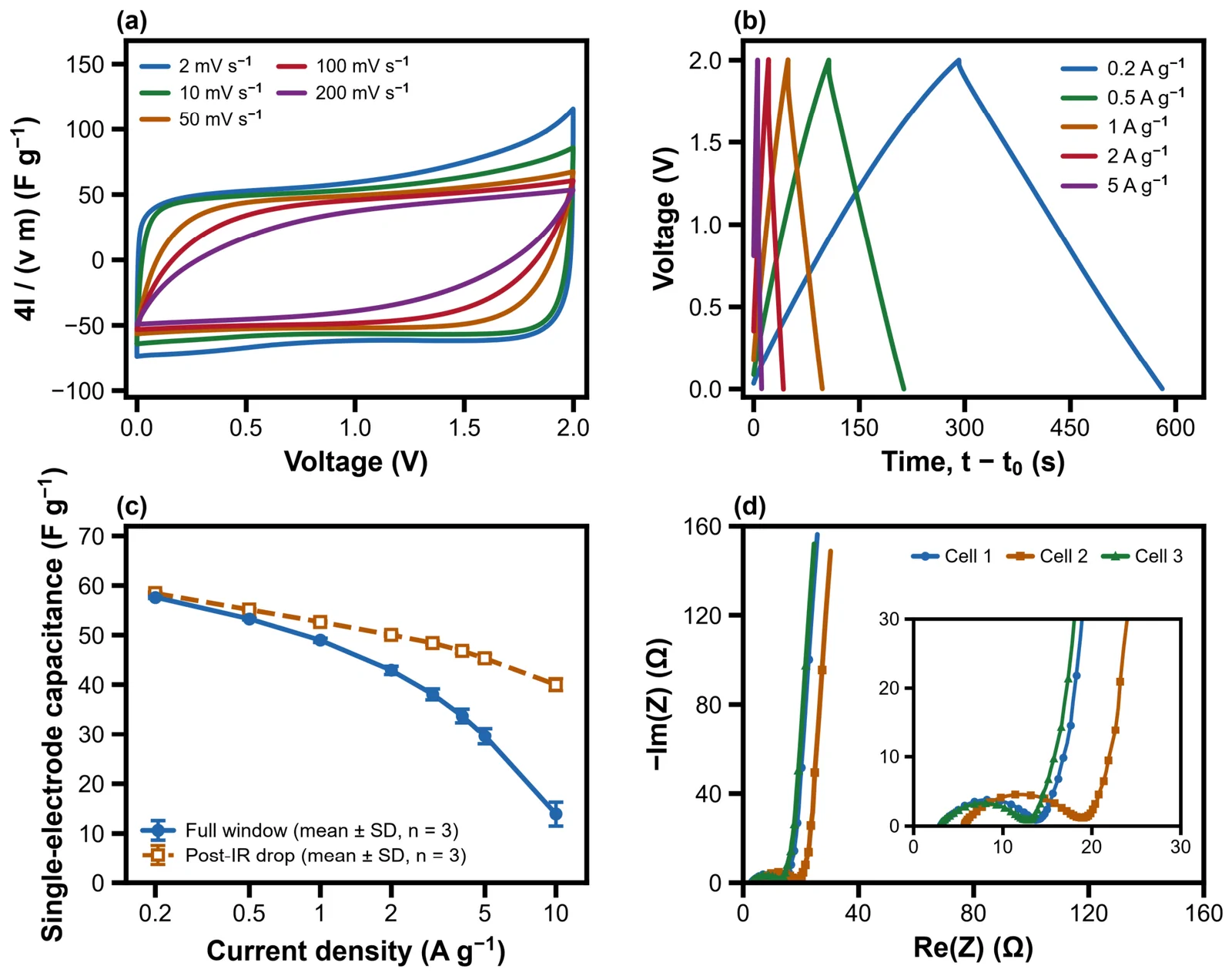
Symmetric Li-CL coin cells in aqueous 10 mol kg^−1^ LiTFSI: (**a**) CVs and (**b**) GCD curves of cell 2; (**c**) specific capacitance vs. current density; (**d**) Nyquist plots.

**Figure 6:**
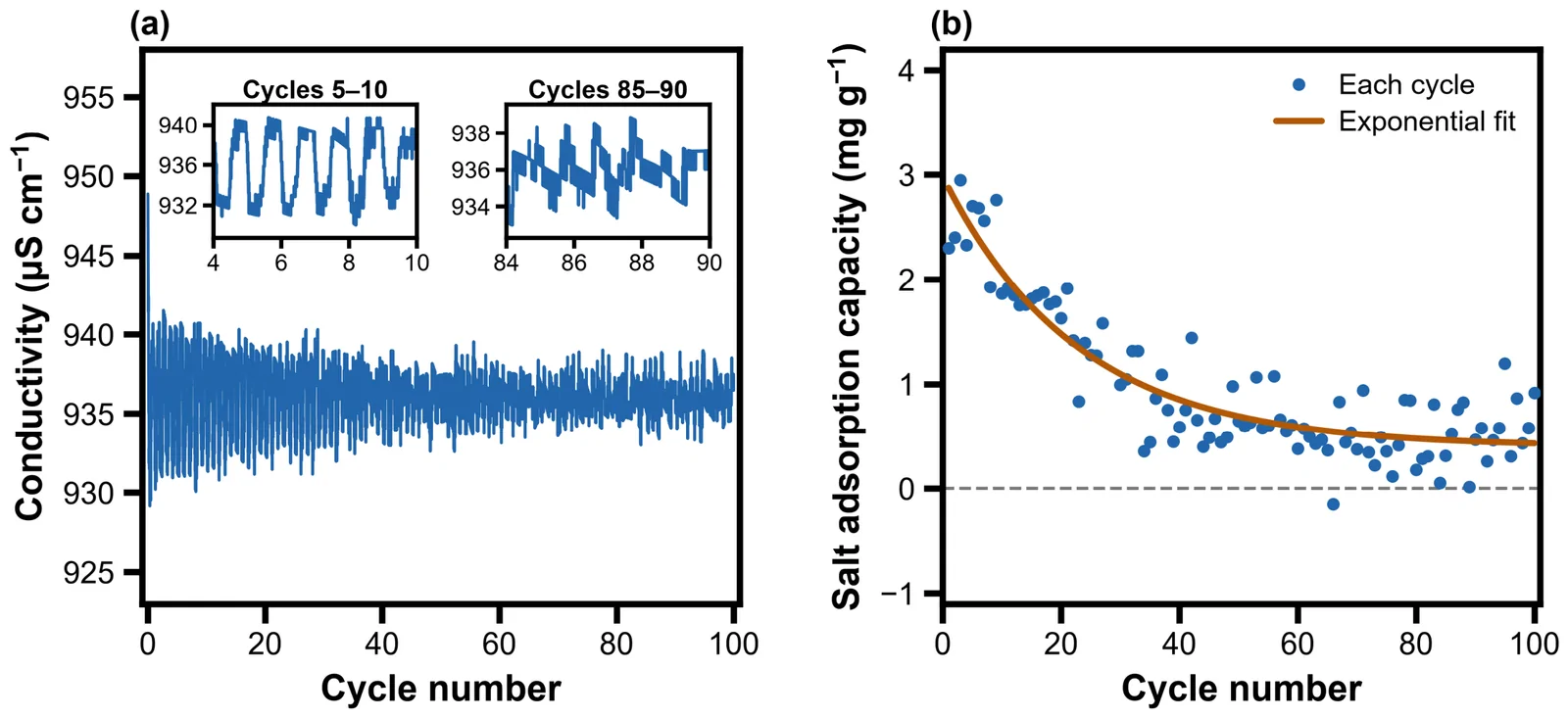
Batch CDI of Li-CL in 10 mmol L^−1^ NaCl: (**a**) baseline-corrected conductivity; (**b**) salt adsorption capacity with a single-exponential fit (line).

**Figure 7:**
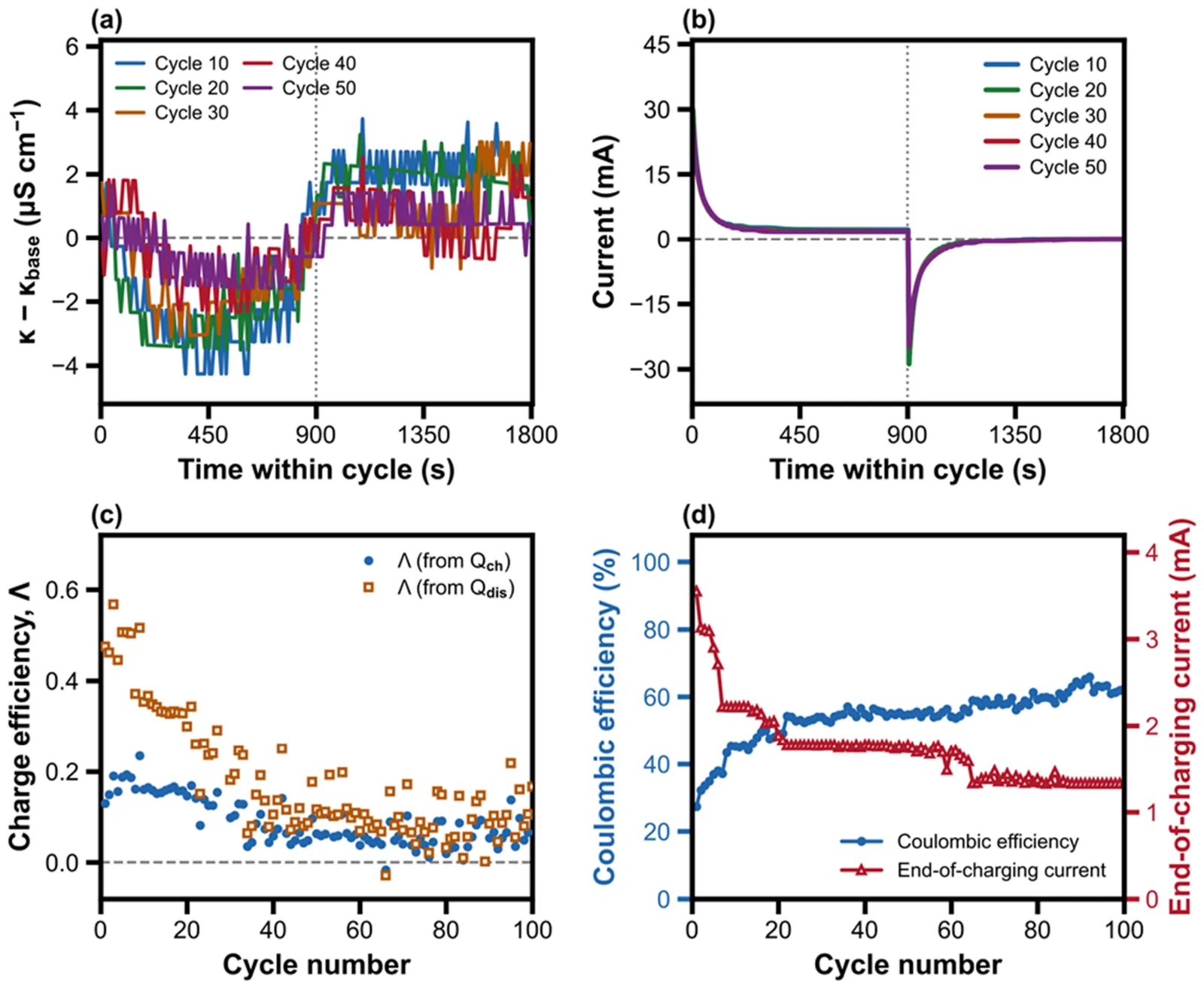
CDI charge characteristics: (**a**) conductivity and (**b**) current transients of cycles 10–50, with the vertical dotted lines at 900 s marking the switch from 1.2 V (adsorption) to 0 V (regeneration); (**c**) charge efficiency; (**d**) coulombic efficiency and end-of-charge current.

**Table 1:** Textural parameters of Li-CL from nitrogen adsorption at 77 K.

Parameter	Value
S_BET_ (m^2^ g^−1^)	342
S_NLDFT_ (m^2^ g^−1^); S_NLDFT_/S_BET_	253; 0.74
Micropore surface area below 2 nm (m^2^ g^−1^)	101
Adsorption-equivalent liquid volume at p/p_0_ = 0.9833 (cm^3^ g^−1^)	0.928
Micropore volume below 2 nm, NLDFT (cm^3^ g^−1^)	0.0565
NLDFT volume above 2 nm including boundary bin (cm^3^ g^−1^)	0.833
Terminal NLDFT bin near 50.6 nm (cm^3^ g^−1^)	0.0600
Modal micropore width (nm)	about 0.9
Volume-weighted median above 2 nm (nm)	22
NLDFT area below 2λ_D_ at 1/10/100 mmol L^−1^ (%)	90/73/38

## Data Availability

The original contributions presented in this study are included in the article/Supplementary Material. Further inquiries can be directed to the corresponding author.

## Supplementary Materials

The following supporting information can be downloaded at: https://www.mdpi.com/article/10.3390/ma19214686/s1, Figure S1: Le Bail fit of Li-C: (a) measured and calculated profiles; (b) difference curve; Figure S2: Voltage-window test: (a-c) CVs of cells 1–3; (d) charge ratio vs. upper cutoff voltage; Figure S3: XRD patterns of (a) Li-C and (b) Li-CL; Figure S4: ATR-FTIR spectrum of Li-CL: (a) 4000–400 cm^−1^; (b) 1800–800 cm^−1^; Figure S5: Two-, four- and five-band Raman fits of Li-CL at four locations; Figure S6: SEM-EDX of Li-CL: (a) SEM image with analysis points; (b–d) C, O and Si maps; (e,f) local spectra; Figure S7: Symmetric Li-CL cell in 6 mol L^−1^ KOH: (a) CVs; (b) GCD curves; (c) specific capacitance vs. current density; (d) Nyquist plot; Figure S8: CVs of (a) cell 1, (b) cell 2 and (c) cell 3 at 2–200 mV s^−1^; Figure S9: GCD curves of (a) cell 1, (b) cell 2 and (c) cell 3 at 0.2–10 A g^−1^; Figure S10: Complex capacitance of the three coin cells: (a) C′; (b) C″; Table S1: Jana2006 Le Bail profile parameters for Li-C; Table S2: GCD results, mean ± sample standard deviation across three coin cells; Table S3: FTIR band assignments for final Li-CL; Table S4: Raman fit results by location; Table S5: Local normalized EDX estimates for the five regions marked in Figure S6a; Table S6: Lithium-citrate carbonization and citrate-assisted CDI studies; Table S7: CDI cycling descriptors of the Li-CL electrode pair (one cell) in 10 mmol L^−1^ NaCl at 1.2 V/0 V, given as mean ± sample standard deviation over the listed cycles. References [12,13,14,38,39] are cited in the Supplementary Materials.
