# Supplementary material for: Development of a Method for Producing Nanoporous Carbon Material from Lithium Citrate
Source: Materials (Basel). 2026 Nov 3;19(21):4686. doi: 10.3390/ma19214686 (PMC13765373; doi:10.3390/ma19214686)
Supplement: Supplementary file 1 [file materials-19-04686-s001.zip › materials-4640461-supplementary.pdf]

Supplementary Information

Development of a Method for Producing Nanoporous Carbon Material from Lithium Citrate

Georgiy Aleksan [1,2], Alexander Zakharov [1], Zhanibek Ayaganov [1], Makhmut Biisenbayev [1,2] and Vladimir Pavlenko [1,2,3,*]

[1] The Institute of Combustion Problems, 172 Bogenbay Batyr St., Almaty 050012, Kazakhstan
[2] Carbonix Group LLP, 43 Nazarbayev Ave., Almaty 050012, Kazakhstan
[3] UNESCO Chair for Sustainable Development, Farabi University, 71 Al-Farabi Ave., Almaty 050040, Kazakhstan
* Correspondence: vladimir.pavlenko@kaznu.edu.kz

XRD profile analysis

The Li-C profile was analyzed by Le Bail decomposition in Jana2006 using Li_2CO_3, Li_2O and a tested LiOH candidate. Table S1 lists the cell parameters and profile statistics. The LiOH candidate was included in the profile model but is not assigned as a confirmed phase. The analysis yields lattice parameters and profile agreement factors; phase mass fractions and crystallite sizes were not determined.

Table S1. Jana2006 Le Bail profile parameters for Li-C. Parentheses give parameter standard uncertainties.

Parameter	Value
Li_2CO_3, C2/c	a = 8.3665(5) Å; b = 4.9793(3) Å; c = 6.2014(3) Å; β = 114.6899° (fixed)
Li_2O, Fm-3m	a = 4.61716(19) Å
LiOH candidate	P4/nmm; a = 3.53 Å and c = 4.34 Å (fixed)
Fitting range and background	10-60° 2θ; five-term Legendre polynomial
Cu Kα radiation	λ_1 = 1.54059 Å; λ_2 = 1.54443 Å; $I_{\alpha2}/I_{\alpha1}$ = 0.497
R_p / R_{wp} / GOF	4.19% / 6.14% / 2.76

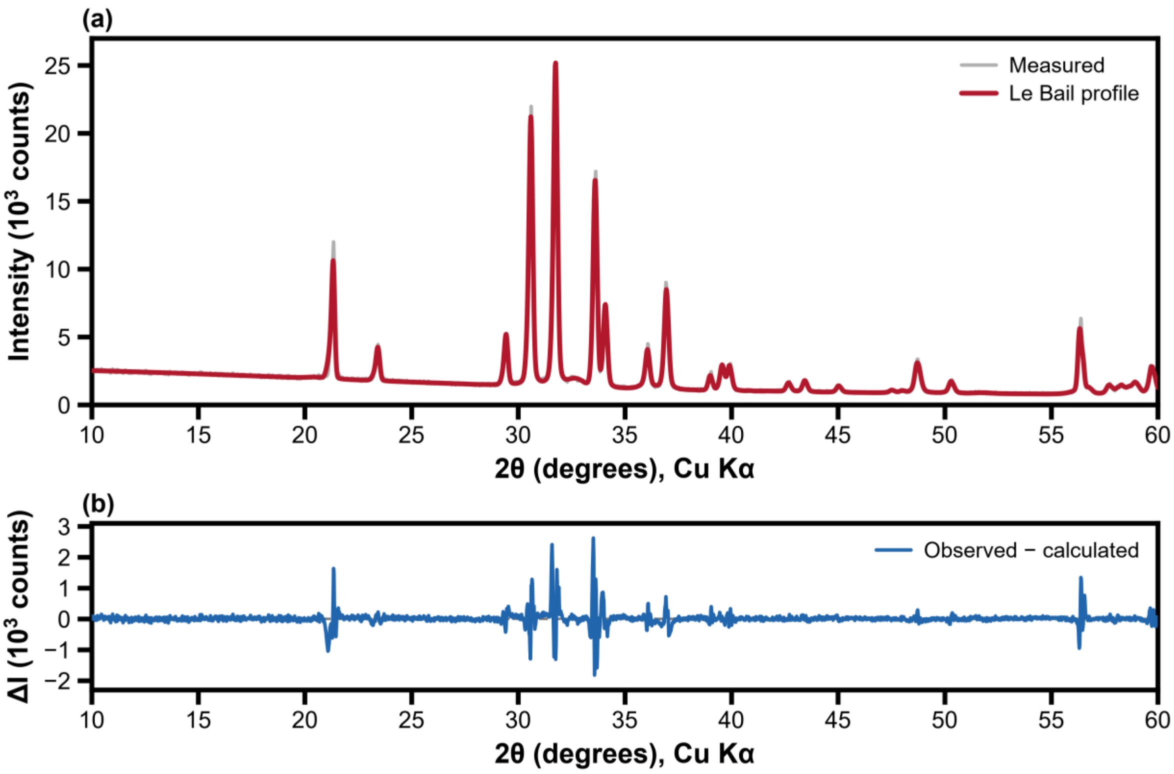

Figure S1. Le Bail fit of Li-C: (a) measured and calculated profiles; (b) difference curve.

Coin-cell assembly and mass normalization

Three Li-CL//Li-CL coin cells were assembled with 12 mm electrode discs (1.131 cm^2), SS316 collectors, ashless cellulose filter paper (red-ribbon grade) and 200 µL of aqueous 10 mol kg^{-1} LiTFSI per cell. The dry composite contained 85 wt.% Li-CL, 10 wt.% Super C45 and 5 wt.% PTFE. The combined composite masses of the two electrodes in cells 1-3 were 10.3, 10.9 and 10.8 mg, corresponding to 8.755, 9.265 and 9.180 mg of Li-CL and to Li-CL loadings of 3.87, 4.10 and 4.06 mg cm^{-2} per electrode. Current densities are normalized by the active mass of one electrode; energy is normalized by the active mass of both electrodes.

GCD calculations and rate response

Each setting contains ten charge-discharge pairs. Cycles 8-10 were averaged within each cell, followed by the mean and sample standard deviation across the three assemblies. The discharge interval starts at the first point with the correct current sign and a current amplitude within 1% of the branch median, and ends at the first zero-voltage crossing, found by linear interpolation. Five transition points were excluded over the full coin-cell dataset, including two in cycles 8–10. Charge and energy were integrated over the measured positive-voltage discharge interval. Figure S9 shows the tenth charge-discharge pairs of the three cells; current density refers to the active mass of one electrode.

Specific capacitance is $C_s = 4Q_d/(m\Delta U)$, using the full nominal window $\Delta U = 2.0$ V. Post-step capacitance instead uses the measured discharge voltage span after current reversal, $\Delta U - \text{Step}$, where Step is the reversal step listed in Table S2, so that $C_{s,post} = 4Q_d/[m(\Delta U - \text{Step})]$. The reversal step is the last charging voltage minus the initial steady-current discharge voltage. Its resistance estimate is $\Delta U_{IR}/(2I)$. Coulombic and energy efficiencies are the discharge-to-charge integral ratios. Discharge energy in Wh kg^{-1} is $\int U(t)|I(t)|dt/(3.6m)$, with m in grams.

Additional records at 20 A g^{-1} contain unresolved discharge steps and do not meet the analysis criterion of at least three discharge points spanning 0.2 V. They are included in the source data but not in the capacitance and energy tables.

Table S2. GCD results, mean ± sample standard deviation across three coin cells.

j (A g^{-1})	C_s (F g^{-1})	Post-step C_s (F g^{-1})	η_Q (%)	η_E (%)	E_d (Wh kg^{-1})	Step (V)
0.2	57.6 ± 0.2	58.4 ± 0.2	98.99 ± 0.05	82.9 ± 0.2	7.61 ± 0.02	0.028 ± 0.002
0.5	53.3 ± 0.2	55.1 ± 0.2	99.25 ± 0.21	81.6 ± 0.4	6.94 ± 0.02	0.068 ± 0.006
1	49.0 ± 0.4	52.6 ± 0.2	99.57 ± 0.08	76.6 ± 1.1	6.12 ± 0.08	0.141 ± 0.015
2	42.9 ± 0.8	50.0 ± 0.3	99.68 ± 0.10	66.3 ± 1.7	4.90 ± 0.16	0.286 ± 0.025
3	38.1 ± 1.1	48.4 ± 0.4	99.72 ± 0.25	57.6 ± 2.1	3.98 ± 0.20	0.429 ± 0.037
4	33.7 ± 1.4	46.8 ± 0.5	99.68 ± 0.06	49.7 ± 2.6	3.19 ± 0.23	0.564 ± 0.047
5	29.6 ± 1.6	45.3 ± 0.8	98.51 ± 1.14	42.6 ± 2.6	2.54 ± 0.25	0.696 ± 0.057
10	13.9 ± 2.4	40.0 ± 1.2	98.25 ± 0.50	18.9 ± 3.3	0.64 ± 0.20	1.312 ± 0.101

Assessment of the working voltage window

The electrochemical voltage window was assessed by a separate CV sequence, recorded after the rate measurements, on the three symmetric Li-CL coin cells in aqueous 10 mol kg^{-1} LiTFSI. Upper cutoffs were increased sequentially from 1.8 to 2.4 V in 0.1 V steps at 2 mV s^{-1}, with the lower cutoff fixed at 0 V. Ten cycles were recorded at 1.8 V and five at each higher cutoff. The last three complete cycles were averaged within each cell, followed by the mean and sample standard deviation across the three cells.

Charge recovery is the ratio of net charge returned on the reverse sweep to charge input on the forward sweep, calculated by current-time integration with the reverse-branch sign inverted. Recovery decreases from 96.83 ± 0.18% at 2.0 V to 92.84 ± 0.28% at 2.2 V and 79.89 ± 0.75% at 2.4 V. The increasing upper-voltage current and declining charge recovery show progressively larger charge losses as the window expands, confirming 2.0 V as an appropriate upper cutoff. Figure S2a-c shows the final cycle at each upper cutoff for cells 1-3, with current normalized by the combined Li-CL mass in both electrodes, and Figure S2d gives the charge ratio as mean ± sample standard deviation across the three cells after averaging the last three cycles per setting; dashed lines mark the 2.0 V cutoff.

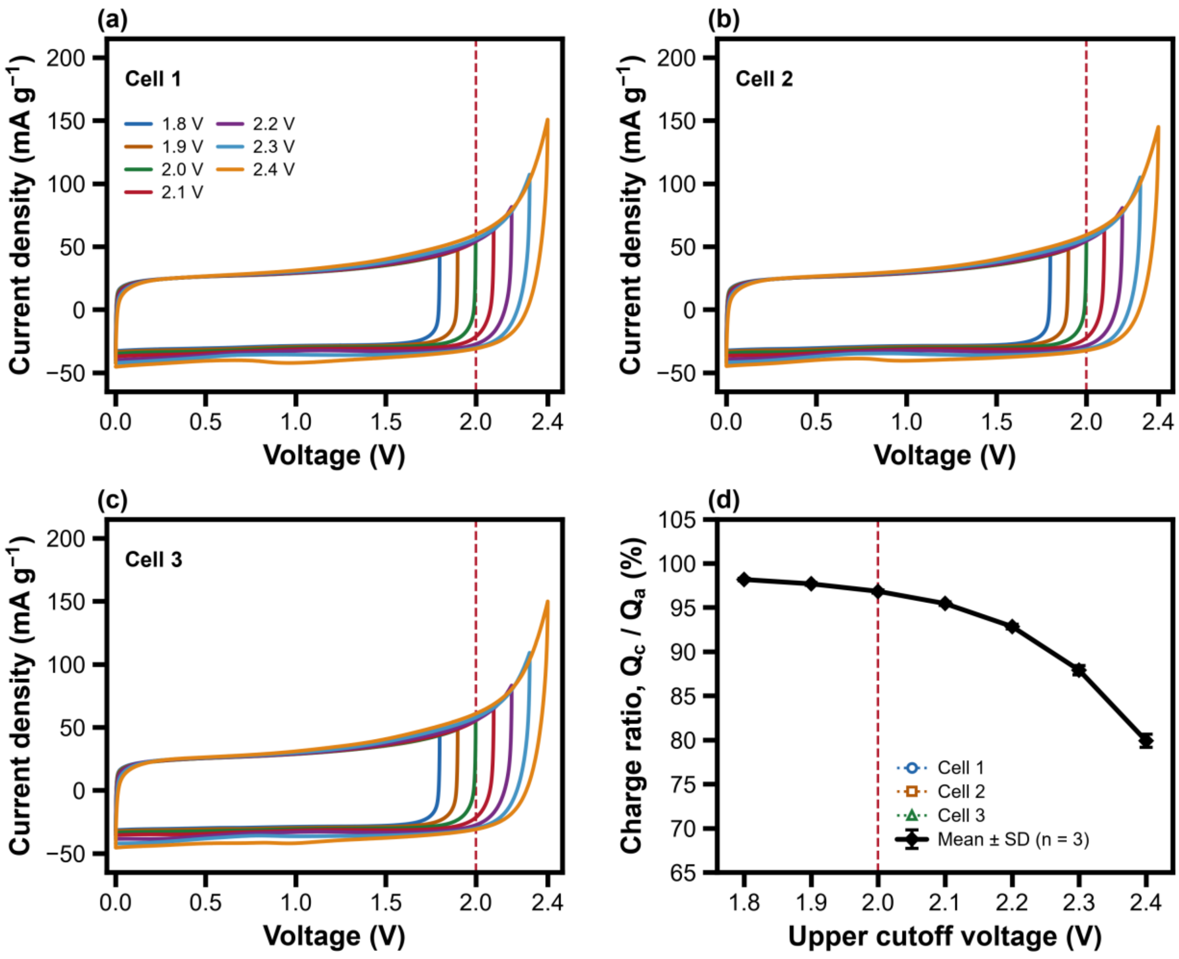

Figure S2. Voltage-window test: (a-c) CVs of cells 1-3; (d) charge ratio vs. upper cutoff voltage.

XRD after leaching and re-annealing

The final Li-CL was measured with Cu Kα radiation at 40 kV and 40 mA, using a Ni filter, 1°/1°/0.2 mm slits, a 5–60° 2θ range, a 0.0188° step and 0.6 s per point. The Li-C scan used 45 kV and 40 mA with the same angular step and counting time. After leaching and re-annealing, the sharp salt reflections are replaced by broad carbon features near 24 and 43° 2θ (Figure S3); each pattern is plotted on its own intensity scale.

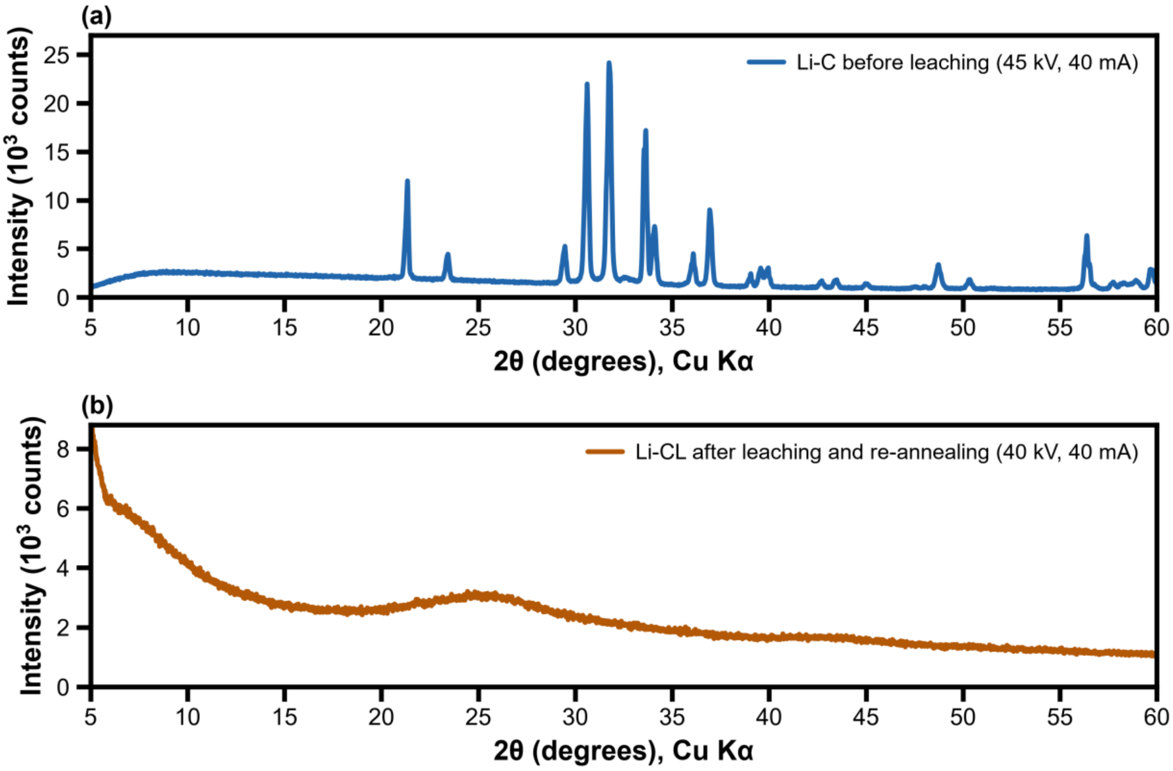

Figure S3. XRD patterns of (**a**) Li-C and (**b**) Li-CL.

FTIR analysis of Li-CL

The ATR-FTIR spectrum was measured on a Bruker ALPHA II equipped with a Platinum single-reflection diamond ATR module, with a spectral resolution of 4 cm^{-1} and 24 co-added scans. Li-CL powder was mixed with KBr at 1:100 by mass and measured on the ATR crystal; no pellet was pressed. Figure S4 shows the full spectrum and an enlarged view of the fingerprint region; the labeled bands are assigned in Table S3.

The band at 1730 cm^{-1} is assigned to C=O stretching in oxygen-containing edge groups. The broad 1566 cm^{-1} band is associated with aromatic C=C skeletal vibrations, with a possible contribution from conjugated C=O groups. The envelope between 1000 and 1300 cm^{-1}, with maxima near 1273 and 1072 cm^{-1}, is dominated by C–O stretching in phenolic, ether and related oxygen-containing groups. The 2921 cm^{-1} feature corresponds to aliphatic C–H stretching [38,39]. Together, the carbonyl and C–O bands show that oxygen-containing functionalities are present in the final carbon after leaching and re-annealing. The broad aromatic response agrees with the disordered carbon framework observed by Raman spectroscopy.

Table S3. FTIR band assignments for final Li-CL.

Band (cm^{-1})	Assignment	Structural interpretation
2921	Aliphatic C–H stretching	C–H groups at non-aromatic sites
1730	C=O stretching	Carbonyl-containing groups
1566	Aromatic C=C skeletal vibrations; conjugated C=O contribution	Conjugated carbon framework
1273	C–O stretching	Phenolic, ether and related oxygenated groups
1072	C–O stretching	Ether/alcohol-type C–O environments

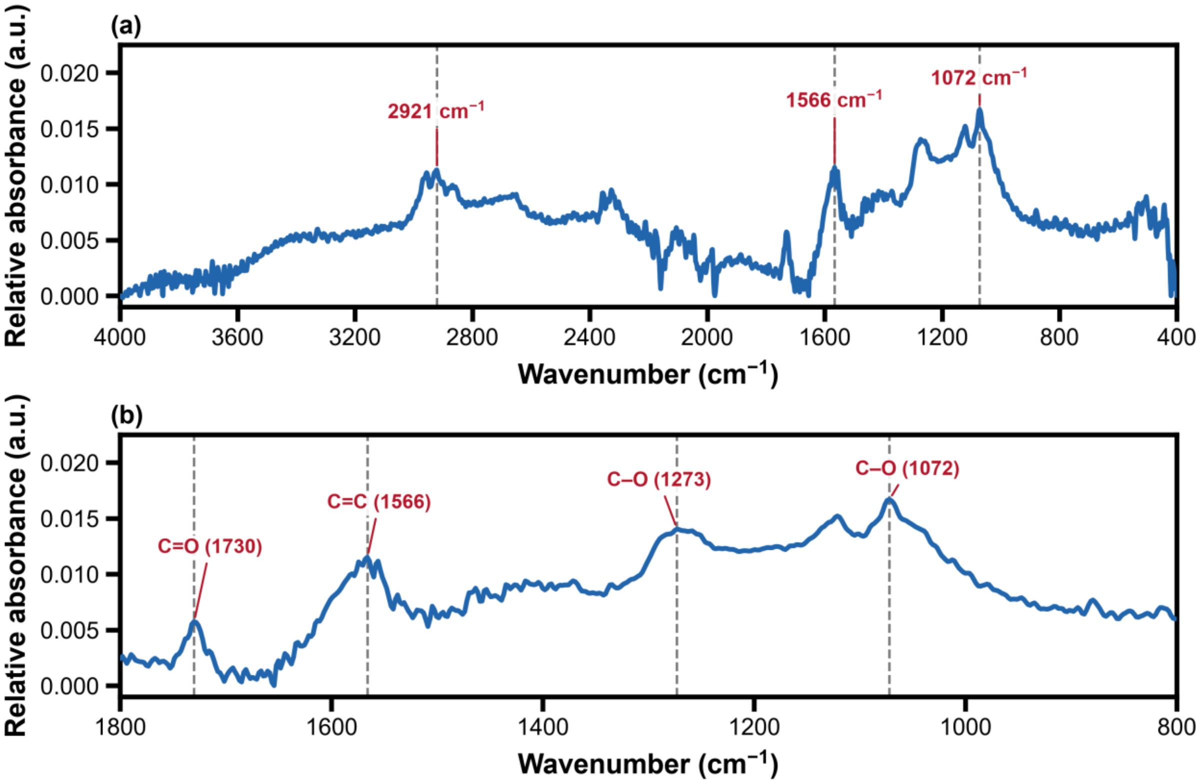

Figure S4. ATR-FTIR spectrum of Li-CL: (**a**) 4000–400 cm^{-1}; (**b**) 1800–800 cm^{-1}.

Raman analysis

Four locations on the final Li-CL were fitted over 900-1800 cm^{-1}. The two-band model contains D1 and G Voigt profiles; the four-band model contains Lorentzian D4, D1 and G profiles and a Gaussian D3 profile; the five-band model adds Lorentzian D2. Band centers were bounded at 1100–1250 (D4), 1310–1410 (D1), 1450–1550 (D3), 1560–1610 (G) and 1605–1650 cm^{-1} (D2). The FWHM was bounded at 30–400 cm^{-1} (15–140 cm^{-1} for D2); the Voigt profiles used a Gaussian σ of 1–180 cm^{-1} and a Lorentzian HWHM of 1–250 cm^{-1}. All fits include a linear background and non-negative component amplitudes. Twelve starting parameter sets were used for each bounded least-squares fit. Figure S5 compares the models at all four locations: gray curves are measured spectra, black curves are total fits, and colored curves are components including the fitted background.

The background slope was bounded between −1 and 1 count per cm^{-1}, and its intercept between zero and the maximum intensity. Component heights were bounded at twice the spectral intensity span; the Voigt area bound was 1500 times that span. Each Voigt profile has four free parameters (area, center, Gaussian σ and Lorentzian HWHM), which together with the two background terms gives the 10 parameters of the two-band

model; the height bounds apply to the Lorentzian and Gaussian components of the four- and five-band models. Isolated narrow spikes were excluded using a five-point median filter with a 20-count threshold, corresponding to the larger of 20 counts and eight scaled median absolute deviations. The same retained points were used for all models: 270, 275, 273 and 276 points at locations 1–4.

Model quality was assessed by the root-mean-square error (RMSE) and Bayesian information criterion, BIC = n ln(RSS/n) + k ln(n), where n is the number of fitted points, RSS the residual sum of squares and k the number of parameters. The two-, four- and five-band models contain 10, 14 and 17 parameters, respectively.

Table S4. Raman fit results by location. D1/G ratios refer to component maxima and integrated areas above the fitted background.

Location	Bands	RMSE (counts)	BIC	D1/G height	D1/G area
1	2	5.41	967.2	1.16	3.82
1	4	3.38	735.6	1.34	3.13
1	5	3.30	739.5	1.45	2.33
2	2	5.56	999.7	1.15	3.95
2	4	3.39	750.1	1.11	2.25
2	5	3.31	754.4	1.90	3.94
3	2	10.29	1328.8	1.06	3.04
3	4	5.58	1016.8	0.87	1.63
3	5	5.46	1022.3	1.47	2.62
4	2	6.42	1082.3	1.10	3.32
4	4	3.76	810.1	0.93	1.72
4	5	3.69	816.0	1.49	2.43

The four-band model has the lowest BIC at all four locations. In the five-band fits, the D2 center reaches 1605 cm^{-1} in every spectrum, and the G center reaches 1560 cm^{-1} at location 1. The boundary-limited D2 and G positions indicate unstable separation of these overlapping bands. The D4 FWHM reaches 400 cm^{-1} at locations 3 and 4. The mean D1/G height ratios are 1.117 ± 0.046, 1.063 ± 0.210 and 1.577 ± 0.218 for two, four and five bands, respectively; standard deviations describe the four measured locations.

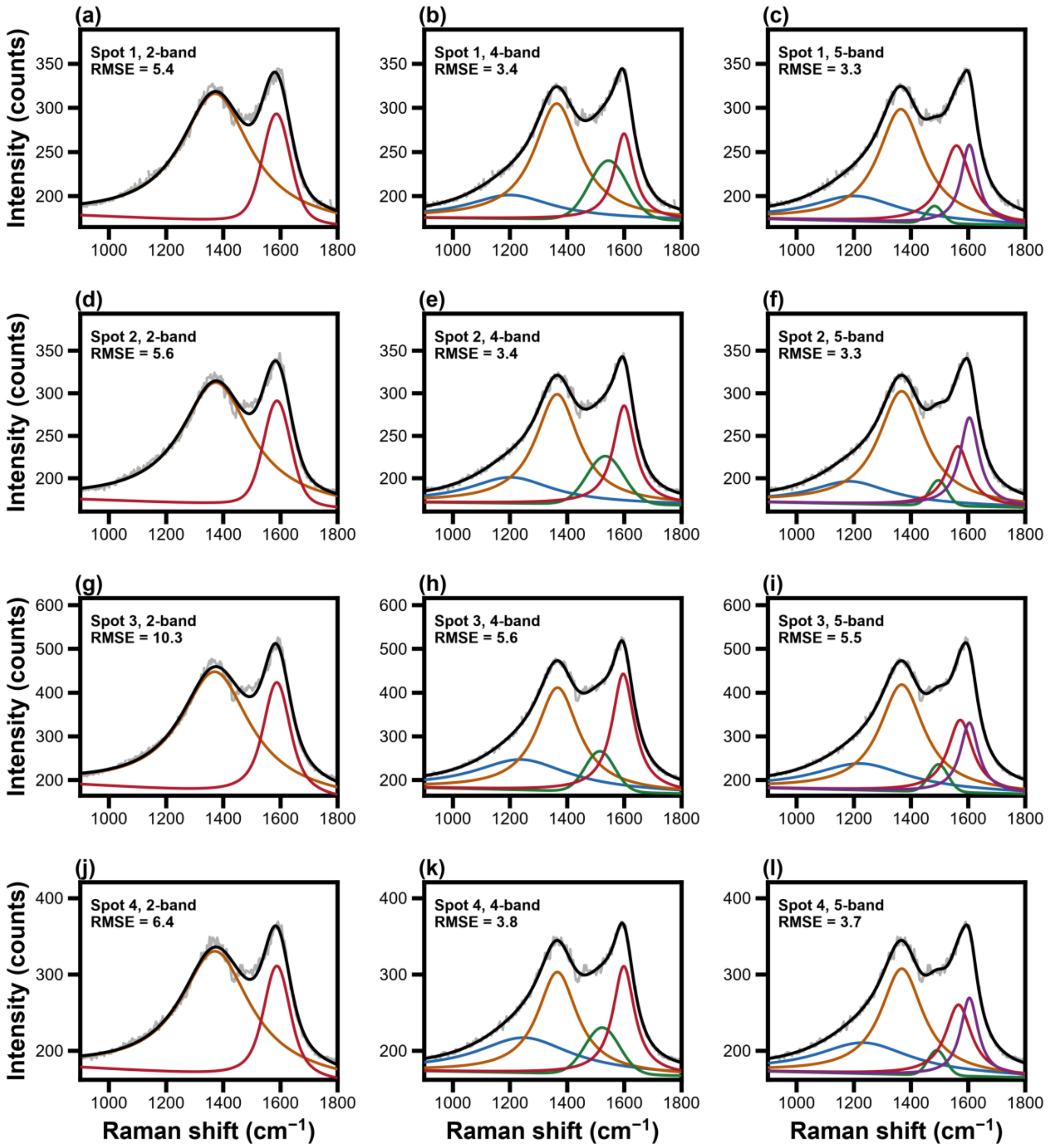

Figure S5. Two-, four- and five-band Raman fits of Li-CL at four locations.

Local composition and elemental distribution

SEM-EDX measurements were performed at 20 kV with a CIQTEK SEM5000Pro microscope (China) and an Oxford Instruments X-Plore 30 silicon drift detector on loosely dispersed Li-CL powder supported on carbon adhesive tape. No platinum coating was applied. The elemental map comprised 512 × 352 pixels and 2048 energy channels, with a live acquisition time of 144.18 s; five local spectra, numbered 2-6 as in the instrument report, were acquired for 23.14–41.08 s.

The local spectra are dominated by C and O. Regions 3-6 yield normalized EDX estimates of 84.40-89.60 wt.% C and 10.01-15.60 wt.% O. A small Na signal occurs in spectrum 3 (0.16 wt.%). The bright elongated inclusion gives 22.55 wt.% Si in spectrum 2 and coincident Si/O enrichment in the elemental maps (Figure S6). Because the precursor and the washing acid are nominally Si-free, this localized inclusion is attributed to external contamination, possibly from the quartz tube reactor or from grinding and sieving. In

Figure S6, the maps show counts summed over 8 × 8 pixels, with local background subtraction for O and Si and independent color scales; spectral intensities are divided by the live acquisition time, and panel (e) shows the low-energy part of the spectra in panel (f). The Si/O maximum in the maps coincides with the inclusion measured in spectrum 2.

Carbon adhesive tape contributes to the C signal, and surface relief and local sampling affect the normalized results in Table S5. These values describe the analyzed regions rather than the bulk powder composition. Weak peaks near 2.1, 9.4 and 11.1 keV coincide with Pt lines; no Pt coating was applied, and their origin was not identified. Residual lithium was not quantified by EDX.

Table S5. Local normalized EDX estimates for the five regions marked in Figure S6a. Values are normalized over the listed elements included in each spectrum's quantification.

Spectrum	C (wt.%)	O (wt.%)	Si (wt.%)	Na (wt.%)
2	62.29	15.17	22.55	n.q.
3	88.30	11.54	n.q.	0.16
4	89.53	10.01	0.46	n.q.
5	89.60	10.40	n.q.	n.q.
6	84.40	15.60	n.q.	n.q.

n.q., element not included in the quantification of that spectrum. Spectrum 2 targets the Si-containing inclusion; spectra 3-6 sample the surrounding carbon-rich regions.

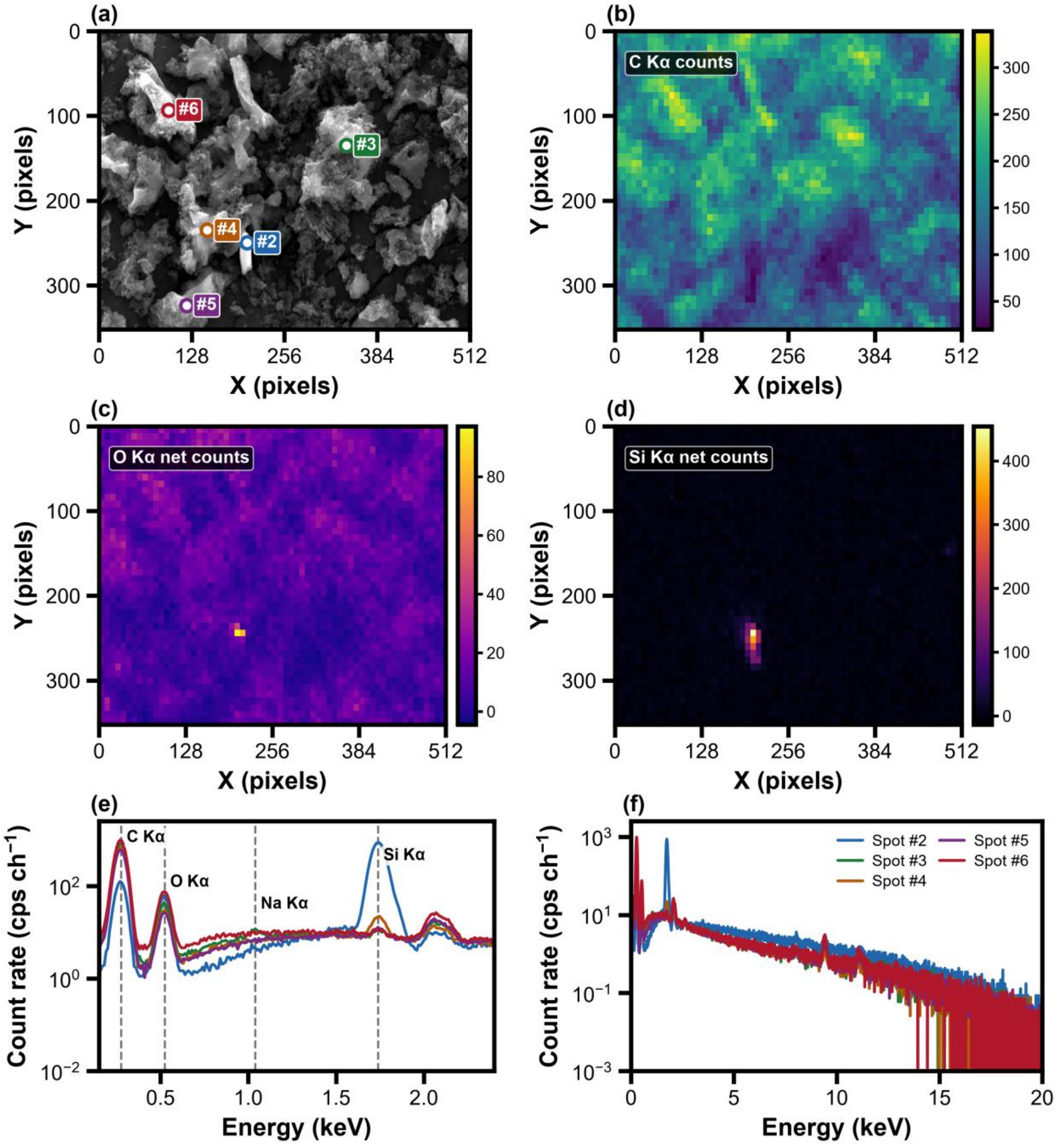

Figure S6. SEM-EDX of Li-CL: (**a**) SEM image with analysis points; (**b–d**) C, O and Si maps; (**e**,**f**) local spectra.

Nitrogen adsorption calculations

BET analysis over p/p_0 = 0.0995-0.3244 (10 points), selected with the Rouquerol consistency criteria and calculated with a nitrogen cross-sectional area of 0.162 nm^2, gives 342 m^2 g^{-1} and C = 52.8. The adsorption-equivalent liquid volume, 0.928 cm^3 g^{-1}, corresponds to 599.3 cm^3 g^{-1} (STP) at p/p_0 = 0.9833, converted with 0.0015483 cm^3 of liquid per cm^3 of gas. Interpolation at p/p_0 = 0.98 gives 0.848 cm^3 g^{-1}, reflecting the steep high-pressure uptake.

The HS-2D-NLDFT cumulative distribution gives 0.0565 cm^3 g^{-1} and 101 m^2 g^{-1} below 2 nm and model totals of 0.890 cm^3 g^{-1} and 253 m^2 g^{-1}. The highest-width bin, at approximately 50.6 nm, contributes 0.0600 cm^3 g^{-1}. Areas below twice the Debye length at 1, 10 and 100 mmol L^{-1} are 90.1%, 72.8% and 38.2%. The adsorption-branch BJH analysis (Harkins-Jura thickness curve, Faas correction) gives a cumulative volume of 0.907 cm^3

g^{-1} over 2.05-116.87 nm. Linear-width distributions were obtained by dividing each incremental volume by its bin width.

Preparation and test conditions in selected studies

Table S6. Lithium-citrate carbonization and citrate-assisted CDI studies.

Study	Preparation and texture	Electrochemical conditions
Li et al. 2021 [14]	Lithium citrate tetrahydrate; 650 °C, 2 h, Ar; 0.1 M HCl, water and ethanol washing. BET 222.5 m^2 g^{-1}; degassing 150 °C, 8 h.	K-ion battery half-cells versus K; 0.8 M KPF_6 in EC/DEC, 0.01–3.0 V.
Shi et al. 2023 [12]	Phoenix-tree leaves, potassium citrate and $NaNO_3$; 800 °C, 2 h, Ar. BET 1912.2 m^2 g^{-1}.	CDI in 500 mg L^{-1} NaCl, 1.2 V, 65 mL, 30 mL min^{-1}; SAC 19.74 mg g^{-1}. Fifty cycles at 15.5 mg cm^{-2}.
Miao et al. 2025 [13]	Glucose, NaCl and potassium citrate; 800 °C, 2 h, N_2; water washing.	Test-carbon cathode/activated-carbon anode; 200 mg L^{-1} NH_4Cl, 1.2 V, 50 mL, 10 mL min^{-1}; 100 cycles.
Present work	Lithium citrate tetrahydrate; 800 °C, 1 h, N_2; 2 M HCl leaching; second 800 °C, 1h anneal. BET 342 m^2 g^{-1}; degassing 350 °C, 12 h.	Three symmetric coin cells in aqueous 10 mol kg^{-1} LiTFSI, 0-2.0 V; 57.6 ± 0.2 F g^{-1} at 0.2 A g^{-1}. Batch CDI in 10 mmol L^{-1} (584 mg L^{-1}) NaCl, 1.2 V/0 V, 100 mL; SAC 2.54 ± 0.28 mg g^{-1} over cycles 1–5 and 0.51 ± 0.30 mg g^{-1} over cycles 80–100.

Measurements in 6 mol L^{-1} KOH

The symmetric cell in 6 mol L^{-1} KOH (Section 2.4 of the main text) was tested over 0–1.0 V. The CV capacitance decreases from 93.0 F g^{-1} at 5 mV s^{-1} to 82.8 F g^{-1} at 50 mV s^{-1}, and the GCD capacitance from 91.5 F g^{-1} at 0.5 A g^{-1} to 80.2 F g^{-1} at 5 A g^{-1} (Figure S7a-c). The impedance spectrum (Figure S7d) shows a depressed high-frequency arc and a nearly vertical low-frequency branch; the phase angle reaches 85.4° at 10 mHz. The line in Figure S7d is a fit to the equivalent circuit L1 + R1 + R2/Q2 + Ma over 300 kHz-10 mHz, with a relative deviation of at most 5%, which gives R1 = 0.090 Ω, R2 = 0.59 Ω and a pore resistance of 0.77 Ω for the distributed element Ma. C′ at 10 mHz is 87.4 F g^{-1}, and the C″ maximum at 0.67 Hz gives τ_0 = 1.5 s. These data were obtained with one cell.

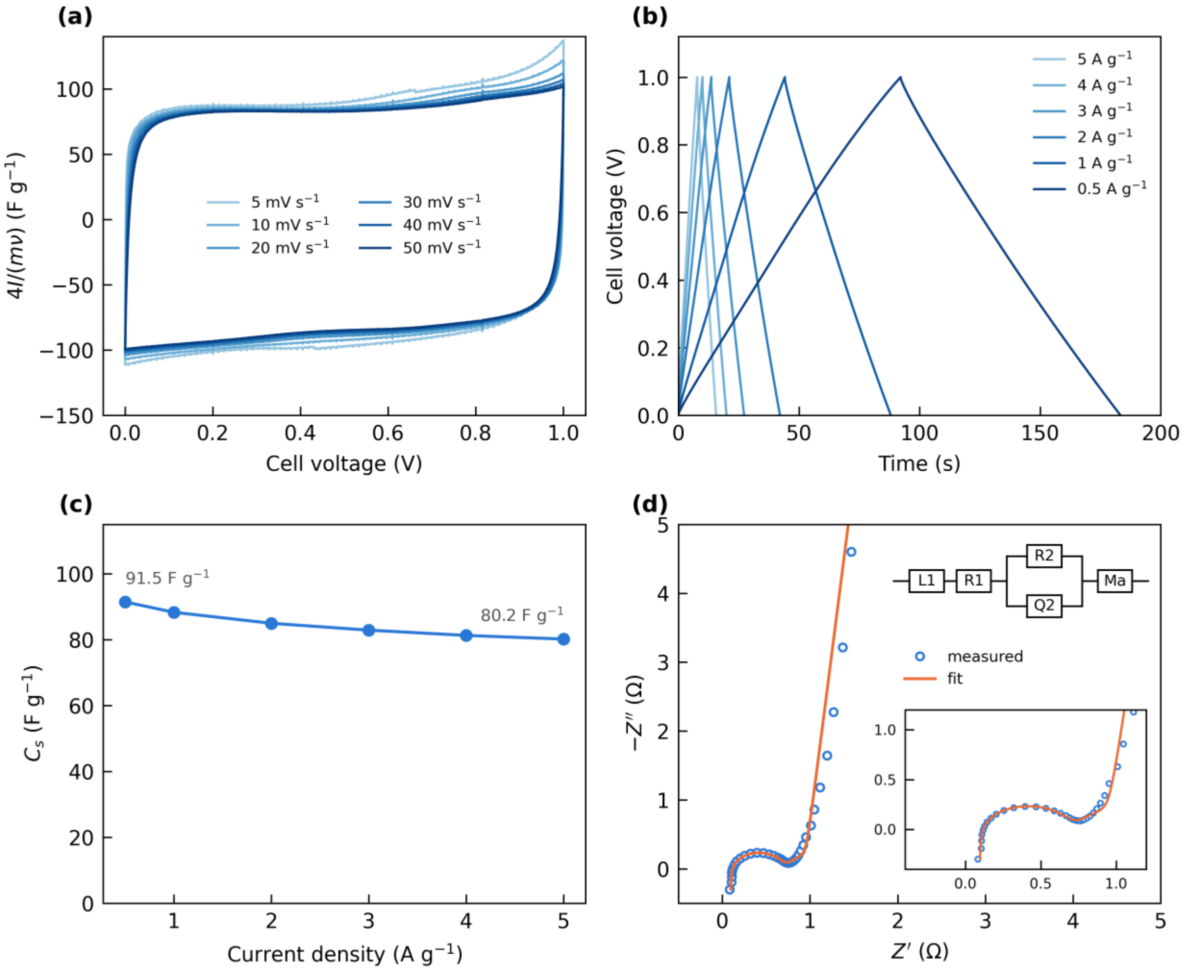

Figure S7. Symmetric Li-CL cell in 6 mol L^{-1} KOH: (**a**) CVs; (**b**) GCD curves; (**c**) specific capacitance vs. current density; (**d**) Nyquist plot.

Cyclic voltammetry

Complete lower-upper-lower voltage cycles were integrated. The CV specific capacitance is $C_{CV} = 2\int|I(t)|dt/(m\Delta U)$, with m the combined active mass and ΔU the cell window. The return-to-forward charge ratio was calculated from the net branch integrals. Results are means of cycles 8-10 within each cell, followed by statistics across the three cells. C_{CV} decreases from 63.2 ± 0.2 F g^{-1} at 2 mV s^{-1} to 47.9 ± 0.5 F g^{-1} at 50 mV s^{-1} and 35.2 ± 1.1 F g^{-1} at 200 mV s^{-1}; the return-to-forward charge ratio is 94.8 ± 1.5% at 2 mV s^{-1} and 98.2–99.8% at 5–200 mV s^{-1}. Figure S8 shows the tenth cycles of the three cells at nine scan rates, with current normalized by the combined Li-CL mass in both electrodes.

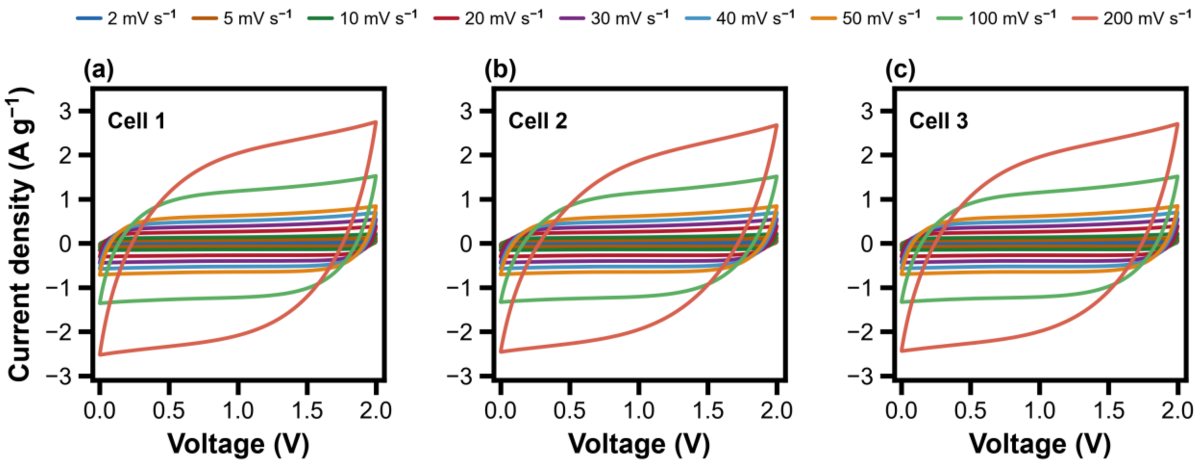

Figure S8. CVs of (**a**) cell 1, (**b**) cell 2 and (**c**) cell 3 at 2-200 mV s^{-1}.

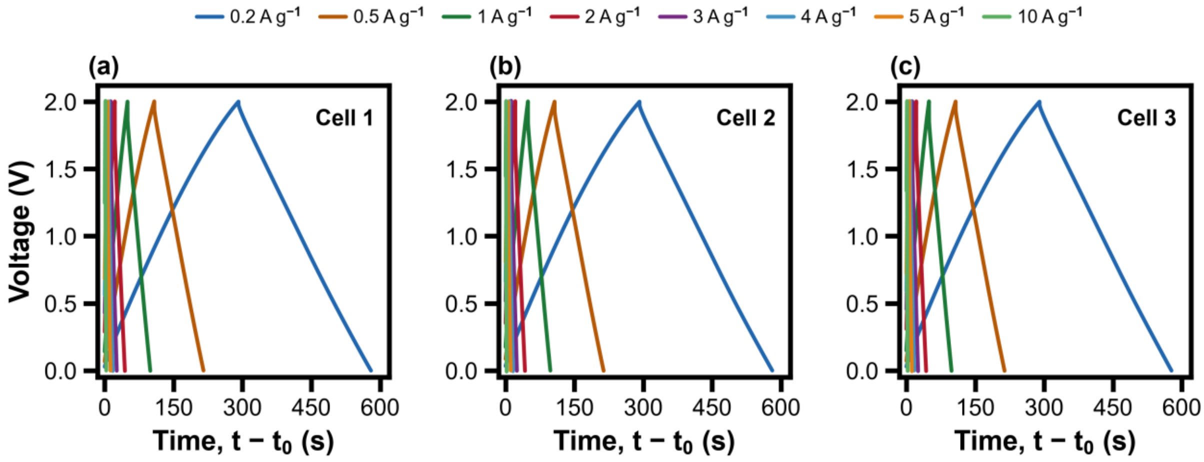

Figure S9. GCD curves of (**a**) cell 1, (**b**) cell 2 and (**c**) cell 3 at 0.2-10 A g^{-1}.

Electrochemical impedance spectroscopy

EIS was measured with a Bio-Logic VSP after CV/GCD. The protocol used a 120 s hold at open circuit, a nominal 10 mV perturbation and 78 frequencies from approximately 500 kHz to 10 mHz. High-frequency real-axis intercepts were obtained by interpolation between adjacent points. Single-electrode complex capacitance is $C^*(\omega) = 4/[m j\omega Z(\omega)] = C'(\omega) - jC''(\omega)$, with $C' = -4Z''/(m\omega|Z|^2)$ and $C'' = 4Z'/(m\omega|Z|^2)$. The relaxation time is $\tau_0 = 1/f_0$, where f_0 is the sampled loss-capacitance maximum. For cells 1-3, the open-circuit voltages were 0.339, 0.339 and 0.365 V, the high-frequency intercepts 3.06, 5.74 and 2.95 Ω, C′ at approximately 10 mHz 45.3, 44.3 and 44.4 F g^{-1}, and f_0 0.079, 0.063 and 0.079 Hz (τ_0 = 12.6, 15.8 and 12.6 s). Figure S10 shows the complex-capacitance spectra; the Nyquist plots are given in Figure 5d.

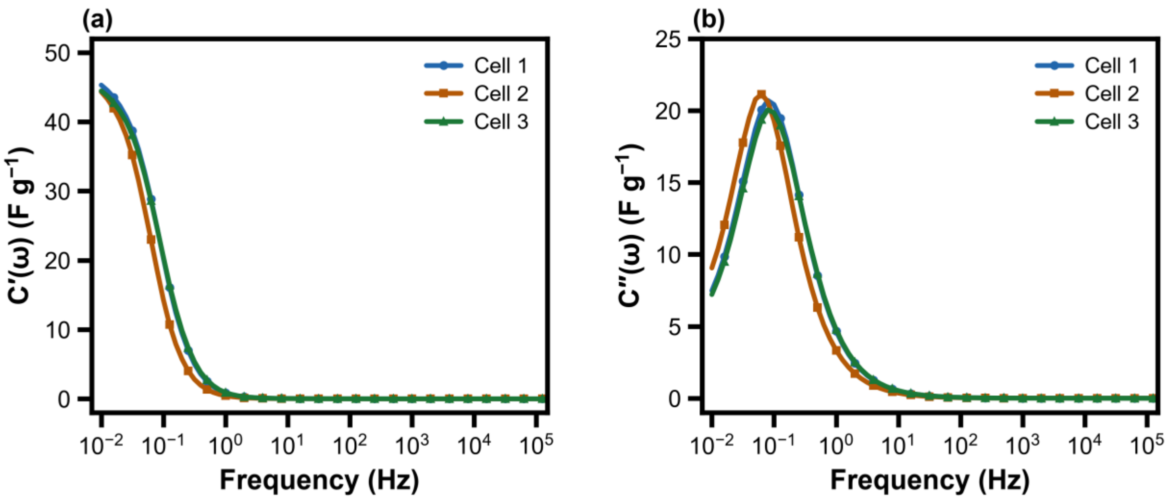

Figure S10. Complex capacitance of the three coin cells: (**a**) C′; (**b**) C″.

Capacitive deionization cycling

The CDI cell was cycled 100 times in batch mode with 100 mL of 10.0 mmol L^{-1} NaCl: 900 s at 1.2 V followed by 900 s at 0 V. The current was recorded every 4 s and the solution conductivity every 10 s. The period of the conductivity modulation, obtained by fitting a square wave to the detrended record, is 1805 s; the programmed cycle is 1800 s, and each recorded voltage step lasted about 903 s. The 1805 s period was used to place the plateau windows. Conductivity recorded during the 10–15 min equilibration before the first voltage step was excluded. The measurements were carried out at room temperature; the solution temperature remained between 23.7 and 25.6 °C.

The baseline was constructed as a shape-preserving piecewise cubic Hermite interpolant through the cycle-mean conductivities placed at the cycle midpoints. The conductivity change of each cycle was obtained from mean values over two plateau windows of the baseline-corrected record, 250-800 s and 1100-1650 s after the start of the 1.2 V step. The windows exclude the switching transients, and averaging over 55 points reduces the effect of the 1 μS cm^{-1} conductivity resolution. As a check, the first-harmonic amplitude of each cycle at the 1805 s period, converted to the equivalent square-wave amplitude, gave mean SAC values within 0.05 mg g^{-1} of the plateau method for cycles 1–20. The concentration conversion uses $\Delta C = C_0 \Delta\kappa / \kappa_0$ with the feed conductivity κ_0 = 990 μS cm^{-1} measured at the start of the equilibration; the conductivity decreased to about 950 μS cm^{-1} by the end of the equilibration, and using 936 μS cm^{-1} would raise all SAC values by about 6%.

Charges were integrated with the trapezoidal rule over each recorded voltage step, which lasted up to 902.96 s. The first recorded point is at 4.0 s, so the current over the first interval was taken equal to its first recorded value. Charge efficiency and the nominal capacitance were calculated as described in the main text, using the combined Li-CL mass of 163.9 mg. SAC is expressed per combined Li-CL mass of both electrodes. Table S7 summarizes the cycle ranges discussed in Section 3.6. The line in Figure 6b is a least-squares fit of $SAC = SAC_\infty + A \exp[-(n-1)/n_0]$ to the 100 cycles with conductivity data (SAC_∞ = 0.40 ± 0.07 mg g^{-1}, A = 2.47 ± 0.12 mg g^{-1}, n_0 = 23 ± 3 cycles, R^2 = 0.83); it describes the trend and is not used to derive any of the reported values.

Table S7. CDI cycling descriptors of the Li-CL electrode pair (one cell) in 10 mmol L^{-1} NaCl at 1.2 V/0 V, given as mean ± sample standard deviation over the listed cycles.

Quantity	Cycles 1–5	Cycles 16–20	Cycles 1–20	Cycles 40–60	Cycles 80–100

SAC (mg g^{-1})	2.54 ± 0.28	1.78 ± 0.09	2.13 ± 0.42	0.68 ± 0.26	0.51 ± 0.30
Q_{ch} (C)	4.26 ± 0.34	3.05 ± 0.03	3.47 ± 0.53	2.75 ± 0.02	2.42 ± 0.07
Q_{dis} (C)	1.40 ± 0.05	1.49 ± 0.03	1.44 ± 0.05	1.51 ± 0.02	1.49 ± 0.03
Coulombic efficiency (%)	33.0 ± 3.6	48.8 ± 1.0	42.4 ± 6.6	55.0 ± 0.8	61.8 ± 2.1
Current at end of 1.2 V step (mA)	3.16 ± 0.24	2.03 ± 0.09	2.43 ± 0.47	1.73 ± 0.06	1.34 ± 0.03
Λ based on Q_{ch}	0.16 ± 0.03	0.16 ± 0.01	0.17 ± 0.02	0.07 ± 0.03	0.06 ± 0.03
Λ based on Q_{dis}	0.49 ± 0.05	0.32 ± 0.01	0.40 ± 0.08	0.12 ± 0.05	0.09 ± 0.05
Nominal C_s (F g^{-1})	28.4 ± 1.0	30.3 ± 0.7	29.2 ± 1.0	30.8 ± 0.4	30.4 ± 0.5
